# Identification, Characterization, and Genomic Analysis of Novel *Serratia* Temperate Phages from a Gold Mine

**DOI:** 10.3390/ijms21186709

**Published:** 2020-09-13

**Authors:** Katarzyna Bujak, Przemyslaw Decewicz, Jerzy Kaminski, Monika Radlinska

**Affiliations:** Department of Environmental Microbiology and Biotechnology, Institute of Microbiology, Faculty of Biology, University of Warsaw, Miecznikowa 1, 02-096 Warsaw, Poland; k.bujak@biol.uw.edu.pl (K.B.); decewicz@biol.uw.edu.pl (P.D.); jerzy.kaminski@student.uw.edu.pl (J.K.)

**Keywords:** bacteriophage, prophage, temperate virus, comparative genomics, *Serratia*, dam-like methyltransferase, non-specific DNA methyltransferase

## Abstract

Bacteria of the genus *Serratia* inhabit a variety of ecological niches like water, soil, and the bodies of animals, and have a wide range of lifestyles. Currently, the complete genome sequences of 25 *Serratia* phages are available in the NCBI database. All of them were isolated from nutrient-rich environments like sewage, with the use of clinical *Serratia* strains as hosts. In this study, we identified a novel *Serratia* myovirus named vB_SspM_BZS1. Both the phage and its host *Serratia* sp. OS31 were isolated from the same oligotrophic environment, namely, an abandoned gold mine (Zloty Stok, Poland). The BZS1 phage was thoroughly characterized here in terms of its genomics, morphology, and infection kinetics. We also demonstrated that *Serratia* sp. OS31 was lysogenized by mitomycin-inducible siphovirus vB_SspS_OS31. Comparative analyses revealed that vB_SspM_BZS1 and vB_SspS_OS31 were remote from the known *Serratia* phages. Moreover, vB_SspM_BZS1 was only distantly related to other viruses. However, we discovered similar prophage sequences in genomes of various bacteria here. Additionally, a protein-based similarity network showed a high diversity of *Serratia* phages in general, as they were scattered across nineteen different clusters. In summary, this work broadened our knowledge on the diverse relationships of *Serratia* phages.

## 1. Introduction

Bacteriophages (phages) are the most abundant and diverse group of biological entities on Earth. They were isolated from every environment inhabited by bacteria [1]. However, our knowledge of phage biology, evolution, and ecology is biased toward phages that infect bacterial strains that are predominantly involved in human diseases and food industry.

Currently, the complete genomic sequences of 25 *Serratia* phages are available in the NCBI Viral RefSeq database. *Serratia* is a Gram-negative, facultative anaerobic, rod-shaped bacteria of the *Yersiniaceae* family of the order *Enterobacterales* [2]. *Serratia* spp. are commonly found in water, soil, plants, the external surfaces of animals, and also in their gastrointestinal tract [3]. *Serratia marcescens* is an opportunistic human pathogen associated with hospital-acquired infections [4,5]. Occasionally, human infections were also described for *Serratia liquefaciens, Serratia rubidaea, Serratia odorifera,* and *Serratia fonticola*. Many antibiotics are not effective against *Serratia* spp. due to their multiple antimicrobial resistances. Therefore, there was a re-emergence of interest in phages as potential therapeutic agents [6].

All known *Serratia* phages belong to the order *Caudovirales*, classified into *Myo*- (twelve phages), *Sipho*- (five), *Ackermann-* (four), and *Podoviridae* (four) (Table S1). Only two *Serratia* viruses were likely temperate—Eta [7] and Parlo [8]. Although the Eta phage produces turbid plaques, none of its encoded proteins are homologous to integrase and the CI repressor-like protein, Eta, therefore was not categorized as classically temperate [7]. On the other hand, Parlo was not experimentally characterized [8]. To the best of our knowledge, all of the above-mentioned *Serratia* phages were isolated from nutrient-rich environments, e.g., raw sewage, fecal matter, wastewater treatment plants, and park and fish ponds, using various clinical or established laboratory strains of *Serratia* spp. as hosts. In contrast, our study describes a *Serratia* virus not related to any of the aforementioned *Serratia* phages that were isolated from distinct oligotrophic environment, the ancient gold and arsenic Zloty Stok mine (Poland), with the use of a strain derived from this niche.

The Zloty Stok mine is a unique place, due to its extreme physico-chemical conditions and exceptional microbial communities, which comprise both heavy-metal-tolerant and psychrotolerant species [9,10,11]. One gallery within the mine, Gertruda Adit, is inhabited by various structurally organized groups of microorganisms, including one that forms mats in the bottom sediments and the another that forms a thick slimy biofilm on the mine walls. Two bacteria isolated from the microbial mats (*Aeromonas* sp. O23A and *Shewanella* sp. O23S), which are among the dominant dissimilatory arsenate reducing strains in this environment, were thoroughly examined, together with their prophages [12,13]. 

Here, we characterize a novel temperate bacteriophage, named vB_SspM_BZS1, isolated from rock biofilms, infecting a local inhabitant of the microbiocenosis, namely, *Serratia* sp. OS31. Additionally, we successfully induce a prophage of *Serratia* sp. OS31 with mitomycin C, named vB_SspS_OS31. Comparative genomic analyses reveal that both these temperate phages are distinct from all other known *Serratia* phages and highlight the uniqueness of BZS1 among known bacterial viruses.

## 2. Results and Discussion

### 2.1. Identification and Characterization of the Temperate vB_SspM_BZS1 Phage

New strain designated *Serratia* sp. OS31 was isolated from a sample of rock biofilms of the Zloty Stok gold and arsenic mine. We succeeded in isolating novel bacteriophage vB_SspM_BZS1, further referred to as BZS1, from the clear zone generated after application of 10 µL of the processed environmental sample on *Serratia* sp. OS31 lawn. Since no enrichment culture was used and the phage could be isolated directly, it could be assumed that the phage titer in the sample was relatively high.

**Figure 1:**
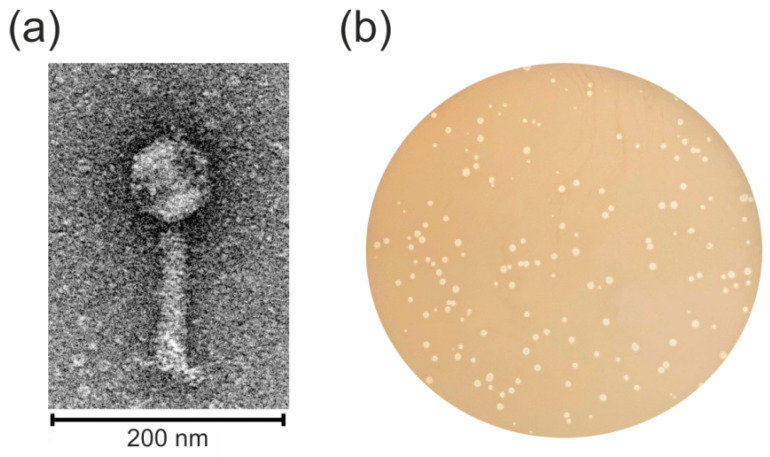
Characteristics of the virion (**a**) and plaques (**b**) of the vB_SspM_BZS1. The scale bar in the transmission electron microscopy (TEM) image represents 200 nm.

Electron micrographs of negatively stained BZS1 virions showed an icosahedral head and a contractile tail (Figure 1a). The average particle had a capsid of approximately 70 nm in diameter, a tail length of approximately 120 nm and was 20 nm in width. Therefore, the virus was morphologically similar to phages of the *Myoviridae* family in the order *Caudovirales*.

Bacteriophage BZS1 formed turbid plaques on a *Serratia* sp. OS31 lawn, with sizes ranging from 0.5 to 3.0 mm in diameter (Figure 1b). Single colonies isolated from the centers of turbid plaques were used to obtain the BZS1 lysogenized host (*Serratia* sp. OS31L_BZS1_). The lysogenic strain was found to be resistant to BZS1 infection. Moreover, the plaques were formed when the naïve parent strain was exposed to the lysogenic strain *Serratia* sp. OS31L_BZS1_, indicating the spontaneous activation of the BZS1 prophage. This lysogenic strain was also tested using PCR reactions with phage-specific primers and the presence of the BZS1 prophage sequence inserted into the *Serratia* sp. OS31L_BZS1_ genome was demonstrated (see below). These results indicated that BZS1 is a temperate phage. BZS1 is the first *Serratia* phage for which the maintenance of a stable lysogeny within the host was revealed. It was also the first temperate *Serratia* myovirus discovered, as Eta and Parlo, if temperate, are classified as *Siphoviridae* and *Podoviridae*, respectively. Eta is not categorized as classically temperate due to the lack of integrase and CI-like repressor geneses and the formation of unstable lysogens [7], and Parlo is not yet experimentally tested [8]. Nonetheless, we have identified integrase (QBQ72150.1) and CI-like repressor (BQ72165.1) encoding genes in the Parlo genome.

### 2.2. Genomic Analysis of the vB_SspM_BZS1 Phage

#### 2.2.1. Identification of the Genome Termini of the vB_SspM_BZS1 Phage

The genome of bacteriophage BZS1 consists of double-stranded DNA with a length of 44,995 bp. Its G + C content (51.6%) was slightly lower than the average for its host genome (the calculated G+C content of 55.3% was determined on the basis of the draft genome of *Serratia* sp. OS31). This was the smallest genome yet of any described functional myovirus of *Serratia*. Ten of the twelve known *Serratia* myoviruses had large genomes (over 150 kb), including a jumbo phage BF with a >350 kb genome [14] (Table S1). The smallest (~68.7 kb) so far included MTx [15] and MyoSmar [16].

The phage termini and the possible packaging mechanism were predicted according to the in silico determination method proposed in PhageTerm [17]. This software was used to analyze raw sequencing reads in the Illumina MiSeq technology data of the BZS1 genome. The PhageTerm program predicted that BZS1 had a linear genomic DNA with 5′ cohesive ends and that the protruding overhang had a nucleotide sequence of GCATTGCGCGCC. This suggests that the BZS1 genome utilizes the cohesive end packaging strategy of λ-like phages [18]. As cohesive ends are covalently joined together in the prophage state, forming a so-called *cos* site, we used two primers to amplify the region containing the putative *cos* site of the BZS1 prophage in the lysogenic strain *Serratia* sp. OS31L_BZS1_. Next, we compared the DNA sequence of this PCR product with those of the corresponding regions of the DNA isolated from free phage particles. The alignment of the sequences of the PCR product and phage ends revealed that the BZS1 prophage indeed contains one 12-bp *cos* site.

#### 2.2.2. Identification of the vB_SspM_BZS1 Attachment Site

tRNA genes are known to be integration target sites for numerous temperate phages [19]. We found that 46 nucleotides downstream of the 3′ end of the putative integrase gene (*phiBZS1_p29*) are complementary to the 3′ end of the tRNA-Lys (CTT) gene of *Serratia* sp. OS31. Therefore, we supposed that this might be a putative phage integration site. To investigate this hypothesis, we used the bacterial chromosomal DNA of the BZS1 lysogenized host *Serratia* sp. OS31L_BZS1_ and PCR assays. PCR primers were designed to amplify the left and right presumed integration flanking regions. PCR fragments of the expected size were successfully obtained. Comparison of the sequencing results of the PCR products showed the expected junction fragments and confirmed that BZS1 integrates into the tRNA-Lys (CTT) gene in the *Serratia* sp. OS31 genome.

#### 2.2.3. Module Analysis of the vB_SspM_BZS1 Genome

The BZS1 genome was predicted to contain 74 open reading frames (ORFs), 44 of which were rightward, and 29 were leftward directed. No gene encoding tRNA was detected in the genome. Both the annotation and organization of the BZS1 genome are provided in Table S2. Function was assigned to 43 ORFs (58% of the total gene number). The remaining 31 ORF-encoded proteins showed similarities with hypothetical proteins that were already described, but were of an unknown function.

**Figure 2:**
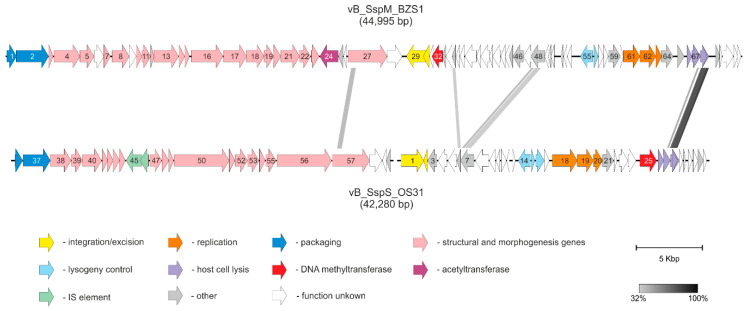
Genome organization and location of homologous proteins of vB_SspM_BZS1 and vB_SspS_OS31 phages. Arrows indicate transcriptional orientation of genes. Selected predicted protein functions are indicated by colored arrowed according to the legend. Numbers on arrows refer to locus tags, i.e., PhiBZS1_pX or PhiOS31_pX, where X refers to gene number in Tables S2 and S3. Additionally, lines connecting BZS1 and OS31 arrows reflect protein sequence similarity according to the scale. For better clarity circular permutation of the vB_SspS_OS31 genome is presented.

The phage BZS1 genome could be divided into at least six functional modules, including integration/excision, regulation (the lysogeny/lysis decision), DNA replication, host cell lysis, DNA packaging, and head and tail morphogenesis (Figure 2).

The integration and excision module of the BZS1 phage was composed of two genes, encoding putative integrase (*phiBZS1_p29*) and excisionase (*phiBZS1_p30*). The predicted integrase belonged to the tyrosine recombinase family [20]. Both PhiBZS1_p29 and PhiBZS1_p30 shared 63% and 74% of their sequence identity with their counterparts in *Salmonella* podovirus P22 (GenBank acc. numbers NP_059584.1 and NP_059585.1, respectively) [21]. It should be stressed that the integration/excision cassette of phage BZS1 was the only segment of the genome that displayed high similarity with the phage genome sequences available in the NCBI database (see Section 2.4.3).

Sequence comparison showed that *phiBZS1_p32* encodes a putative type II N6-adenine DNA methyltransferase (m^6^A MTase) that could be classified to the MT-A70 family m^6^A MTases. Phylogenetic analysis revealed that the MT-A70 (also known as METTL3) superfamily consists of four subfamilies, among which one is a small group of prokaryotic m^6^A MTases [22]. The representative of this group is the Dam-like enzyme of transposon Tn1549, conferring vancomycin resistance in *Enterococcus* spp. [23]. The Dam-like activity, i.e., the introduction of Gm^6^ATC methylation in *Gammaproteobacteria*, provides markers to DNA replication, mismatch repair, and the regulation of gene expression [24]. Dam enzymes of commensal and pathogenic strains, e.g., *E. coli, Vibrio, Salmonella, Haemophilus*, or *Yersinia*, were extensively investigated experimentally [25,26,27]. Dam mutants of *S. marcescens* showed an increased mutation frequency upon exposure to UV radiation [28]. Genes encoding Dam-like proteins are also present in numerous bacteriophages, including P1 [29], VT-2 [30], the T-even coliphages [31], *Haemophilus* HP1 [32], and *Aeromonas* PhiARM81mr [33], but the function of phage-encoded Dam MTases in the phage life cycle is still not well understood.

PhiBZS1_p32 showed 41% sequence identity with the abovementioned M.EfaBMDam (GenBank acc. no. AAF72345.1) of *Enterococcus faecalis* transposon Tn1549. In order to determine whether GATC sequences might represent a substrate for PhiBZS1_p32, we isolated the pET_PhiBZS1_p32 plasmid DNAs from IPTG-induced and non-induced cultures of the *E. coli* ER2929 Dam^−^ mutant strain, lysogenized with the λDE3 element. The plasmid DNAs isolated from the induced cultures of the *E. coli* ER2929 Dam^−^ strain were resistant to cleavage with MboI (inhibited by m^6^A methylation) but were sensitive to DpnI (requires adenine methylation of GATC sites for cleavage), and were also sensitive to cleavage with various other enzymes that are sensitive to m^6^A methylation (e.g., HinfI and Hin1II) used as controls. The pET_PhiBZS1_p32 DNA isolated from the non-induced cultures was susceptible to all restriction enzymes used, except DpnI (Figure S1a). Based on these results, we concluded that the sequence specificity of PhiBZS1_p32 was GATC.

It should be stressed that phage- and host-encoded Dam proteins do not share sequence similarity and belong to different classes of DNA MTases (β and α, respectively) [34], thus are not closely related. Therefore, the BZS1-encoded GATC-specific DNA MTase could be considered to be an example of the convergent evolution between the virus and its host enzyme, regarding their sequence specificity.

Interestingly, we identified homologs of PhiBZS1_p32, thus, members of MT-A70 family in the genomes of various viruses, e.g., *Escherichia* phage HK639 (GenBank acc. no. YP_004934065.1, 75% sequence identity), *Klebsiella* phage ST13-OXA48phi12.5 (QEA09481.1), and *Cronobacter* phage phiES15 (YP_006590010.1, 70% sequence identity). In all these phage genomes, the MTase genes were found downstream of the integrase gene, similar to BZS1.

A temperate bacteriophage has both lytic and lysogenic cycles. Lambda phage and its relatives employ products of repressor genes such as CI, that act as genetic switches to control the balance between these two growth states [35]. Activated RecA, the mediator of the host SOS response to DNA damage, causes inactivation of the repressor by stimulating the repressor’s nascent autocleavage activity, allowing phage induction and the expression of early phage genes [36,37]. The predicted CI repressor-like protein of BZS1 is encoded by the *phiBZS1_p55* gene. It belongs to the XRE family of transcriptional regulators (COG2932), and its amino-terminal region consists of a helix-turn-helix domain (HTH, pfam12844), while its carboxyl-terminal region contains the S24 signal peptidase domain (pfam00717) that is typical for RecA-stimulated autocleavage. Thus, the overall organization of PhiBZS1_p55 is similar to other lambdoid bacteriophage repressors. The protein has 58% sequence identity with the putative prophage repressor of the Stx1-converting *Escherichia* phage SH2026Stx1 (GenBank acc. no. AVO22784.1). The divergently transcribed neighboring gene, *phiBZS1_p56*, encodes a putative Cro homologue. It also possesses an HTH domain (pfam1381) and is similar to its functional counterparts of *Salmonella* phages Fels-1 (GenBank acc. no. YP_001700550.1, 54% sequence identity) and ST64B (NP_700412.1, 53% sequence identity).

The PhiBZS1_p61 protein exhibits a significant sequence similarity with several proteins described in the NCBI database as phage replication proteins, e.g., *Klebsiella* phage ST15-VIM1phi2.1 (GenBank acc. no. QBP28246.1), *Pectobacterium* phage ZF40 (YP_007006923.1), and *Salmonella* phage S107 (AXC39935.1). However, a region of sequence identity is limited almost exclusively to the N-terminal half of the protein where the HTH-domain (pfam13730) was identified (1-160 aa). The other half of the PhiBZS1_p61 protein showed no significant similarity to any viral protein. It is possible that two adjacent genes of *phiBZS1_p62-63*, encoding a putative HNH endonuclease (pfam06147) and a RusA-like crossover junction of endodeoxyribonuclease, respectively, are also involved in the replication process of BZS1. The RusA protein of *E. coli* is a DNA structure-specific endonuclease that resolves Holliday junction intermediates formed during DNA replication, recombination, and repair, by introducing symmetrical paired incisions 5′ to CC dinucleotides [38]. It was shown that the RusA protein was also encoded by the r1t phage of *Lactococcus lactis* [38] and the defective prophage DLP12, and rusA-like sequences were associated with prophage sequences in several bacteria [39].

Lysis of Gram-negative hosts was shown to require holins and endolysins that attack the cytoplasmic membrane and peptidoglycan, respectively. Additionally, Rz-Rz1-like protein pairs, referred to as the spanin complex (also annotated as endopeptidases), which disrupt the host outer membrane (OM) and participate in cell lysis during virus exit were identified in nearly all phages of Gram-negative hosts [40,41]. A cluster of BZS1 genes involved in the bacterial lysis encoding holin (PhiBZS1_p66), lysozyme (PhiBZS1_p67), and two endopeptidases (PhiBZS1_p68 and _p69) was identified. All protein products of these genes possess at least one predicted transmembrane domain. PhiBZS1_p67, classified as a glycoside hydrolase (pfam00959), showed high similarity to the lysozymes encoded by *Pectobacterium* phage ZF40 (GenBank acc. no. YP_007006943.1, 78% sequence identity), *Yersinia* phage PY54 (NP_892107.1, 75% sequence identity) [42], and several *Klebsiella* and *Escherichia* phages.

Interestingly, *phiBZS1_p65*, the gene preceding the lysis cluster, encodes a putative homolog of bacterial RcsF, a component of Rcs system (Rcs stands for regulator of capsule synthesis), i.e., an OM lipoprotein that functions as an envelope stress sensor that is capable of mounting a protective response, when damage occurs in the peptidoglycan or in the OM [43,44]. After activation, Rcs induces stress-related mechanisms, including the synthesis of extracellular polysaccharides, which form a protective capsule. It is also used in biofilm formation [45]. The presence of *phiBZS1_p65* in the BZS1 genome is very intriguing. First of all, it appears to be of bacterial origin, as we did not find similar proteins in any other virus. Moreover, its location in the BZS1 genome, upstream of the lysis cassette, might suggest that it affects the timing or efficiency of the host lysis during the phage infection.

It is well established that tailed dsDNA bacteriophages use the same packaging machines. After the replication of genomic DNA, the replicating concatemeric chromosome DNA is cut into genome-sized lengths by the endonuclease activities of terminase proteins and is subsequently packaged into the preassembled capsid, relying on the ATPase activity of these enzymes [46]. The packaging machine usually consists of two components, namely, a small terminase (TerS) and a large terminase (TerL). The putative product of the *phiBZS1_p01* gene possesses an HTH-domain (pfam13518) and thus likely encodes TerS. A BLASTP search in the NCBI virus database revealed no homologs for PhiBZS1_p01. A putative TerL (PhiBZS1_p02) shares similarity with a TerL of *Synechococcus* phage S-CAM8 (GenBank acc. no. AET72686.1, 46,4% sequence identity) and several TerL proteins identified in assembled genomes of uncultured Mediterranean viruses [47].

Gene clusters encoding phage structural proteins are typically located adjacent to the DNA packaging module. The predicted structural gene cluster of BZS1 covers similarly oriented ORFs from *phiBZS1_p03* (predicted as encoding a phage head–tail adapter) to *phiBZS1_p27-28* (initially predicted as encoding a tail fiber and a hypothetical protein, respectively). Interestingly, PhiBZS1_p27-28 shares significant sequence similarity with only two structural proteins of the cold-active siphovirus VW-6B of *Pseudomonas fluorescens* (GenBank acc. no. ATN93650.1, 32.48%, and ATN93649.1, 26.29% identity, respectively). Moreover, within the putative tail fiber protein, PhiBZS1_p27, an acetyl esterase (deacetylase) domain (pfam03629; 99.9% probability) was identified, based on HHpred searches. A conserved protein domain that carries acetyl esterase was found to be associated with tail spikes of all Vi phages that utilize the Vi capsular antigen of *Salmonella enterica* serovar Typhi as a receptor [48]. Vi is a linear, acidic homopolymer of α-1,4-linked N-acetylgalactosaminuronate, with variable O-acetylation at the C-3 position [49]. It was suggested that the acetyl esterases of Vi phages directly target the acetyl modification of the sugars of the capsule itself, which might allow these phages to infect *S. enterica* ser. Typhi [48]. Another example of virion-associated esterase is a baseplate protein of the *Salmonella* c341 phage, but in this case the enzyme deacetylates the O-antigen of the lipopolysaccharide (LPS), rather than the acetyl moiety of the capsule polysaccharide chain [50].

To obtain a more comprehensive view of the BZS1 structural region, SDS-PAGE (Figure S2) and mass spectrometry analyses of the capsid proteins were carried out. Proteomic characterization of the virion particles allowed for identification of 13 BZS1-encoded products (PhiBZS1_p07-08, _p11, _p13-14, _p17-22, and p_27-28, respectively). Among them, one polypeptide, BZS1_p28, initially annotated as a hypothetical/uncharacterized protein (and as mentioned above, with only one homolog in Viral database), could now be described as virion-associated.

Surprisingly, in the region between *phiBZS1_p23* and *phiBZS_p27*, but in the opposite orientation to structural genes, there are three additional genes. PhiBZS1_p24, with a conserved domain of the acyltransferase family (pfam01757; COG1835), shows 26% sequence identity to O-antigen acetyltransferase (Oac) of bacteriophage Sf6 of *Shigella flexneri*, which uses O-antigen of its host as a primary receptor [51]. Consequently, the phage-encoded O-antigen acetylase causes O-serotype conversion of Sf6 lysogens [52], which prevents phage Sf6 adsorption to these cells. Notably, O-antigens are a major component of the surface LPSs of Gram-negative bacteria and allow them, for example, to escape the host’s immune system [53]. Lysogenic bacteriophages often encode enzymes that modify or degrade the structures of LPSs, thereby resulting in altering (serotype conversion) the bacterial cell surface, e.g., Sf6, as mentioned above. Interestingly, the serotype conversion *oac* gene of the Sf6 phage is transcribed in the opposite orientation to its head and tail genes, similar to the arrangement observed in BZS1. This raises a presumption that *phiBZS1_p24* might be actively transcribed from a prophage. 

Although it is highly speculative, we suppose that BZS1 targets the O-antigen of its host LPS and uses the deacetylase activity of PhiBZS1_p27 to modify this receptor to enter the cell. On the other hand, during lysogeny, the acetylase activity of PhiBZS1_p24 (which modifies O-antigen) is beneficial for a prophage, providing protection for the host cells, against attack by superinfecting phages that recognize O-antigen as a receptor.

It should be noted that in the region between a *phiBZS1_p24* putative acetyltransferase and tail fiber genes, there are two other ORFs that are divergently oriented with respect to the structural genes. PhiBZS1_p25 was predicted to contain an HTH motif, and for PhiBZS1_p26, the TMpred program predicted the presence of a single C-terminal transmembrane domain. We have no clue about the role of these proteins. Nonetheless, PhiBZS1_p25 showed a 47% sequence identity with hypothetical protein Pkon1_p72 (GenBank acc. no. AZV00301.1), encoded by *Paracoccus* phage vB_PkoS_Pkon1 [54], which is adjacent to the putative XRE-family transcriptional regulator Pkon1_p71 (GenBank acc. no. AZV00300.1). However, Pkon1_p71 does not exhibit any significant similarity with PhiBZS1_p25 and the location of the *pkon1_p71* and *pkon1_p72* genes in the center of the opposite oriented host cell lysis gene cluster is different than in the case of BZS1. On the other hand, three pairs of adjacent genes, encoding proteins almost identical to PhiBZS1_p25-26, are present in *Serratia proteamaculans* 336X (QGH59474.1-QGH59475.1, QGH60159.1-QGH60158.1, and QGH64144.1-QGH64145.1). There is always a gene encoding acetyltransferase adjacent to each of them, but PhiBZS1_p24 only shares significant similarity with QGH59476.1 (97% identity). These three *S. proteamaculans* 336X gene clusters are arranged in opposite orientations to other genes and are parts of putative prophage sequences. We also identified three similar gene clusters in other *Serratia* spp. genomes, e.g., the UMH6, 1D1416, and AR_0130 strains, which suggests cooperation or dependent components of these clusters.

The BZS1 genome possesses a few more peculiar genes. A putative product of the *phiBZS1_p70* gene is similar to the theta subunit of DNA polymerase III (pfam06440), which is suggested to enhance the proofreading activity of the epsilon subunit. PhiBZS1_p70 showed 67% sequence identity with the Hot (meaning the homologue of theta) of the P1 phage, which can substitute for the *E. coli* DNA polymerase III theta subunit [55]. The location of *phiBZS1_p70* in the BZS1 genome, in a group of late genes, is similar to the Hot P1. Nonetheless, it was demonstrated that the P1 Hot is actively expressed in the lysogenic stage, as well as in both early and late stages of a lytic cycle, and it was hypothesized that the Hot might affect the host replication machinery to benefit the overall phage replication [56]. Located downstream, *phiBZS1_p71* encodes a hypothetical protein containing a zinc-ribbon motif (pfam10571) and a putative protein product of *phiBZS1_p72*, showing similarity to phage minor tail proteins. However, it is possible that the function of the latter protein is other than structural, because *phiBZS1_p72* is not clustered with structural genes, suggesting a different expression timing. Interestingly, its position in the genome (upstream of a small unit of terminase genes in circular genomes) seems to be conserved. PhiBZS1_p72 homologs encoded by the *Dinoroseobacter* phage vB_DshS-R4C (GenBank acc. no. QDF14297.1) and *Pseudomonas* phage vB_Pae_CF165a (QBI77962.1) occupy the same position in genomes. 

We also identified the LexA DNA-binding motif upstream of the *phiBZS1_p37* gene, which was annotated as a hypothetical protein. Although we do not know what function it performs, we can predict that its expression is regulated by LexA. It should be noted that homologous genes in other phages (e.g., *Salmonella* phages SE16, 29485, SF3, and *Klebsiella* phage ST13-OXA48phi12.1), are found upstream of integrase genes, like in BZS1.

### 2.3. Genomic Analysis of the vB_SspS_OS31

The BZS1 host (*Serratia* sp. OS31) was exposed to mitomycin C in order to determine whether it carries any active prophage. This approach resulted in the induction of a phage named vB_SspS_OS31 (OS31 in short). As OS31 was another classical temperate *Serratia* phage, it was included in the analysis.

The DNA of the mitomycin C-induced OS31 phage was isolated and sequenced. Later, the identical prophage sequence was identified in the draft genome of *Serratia* sp. OS31. Additionally, the draft genome of this bacterium was searched for putative prophage sequences but only the OS31 prophage was revealed.

Sequence analysis showed that the genome of OS31 was 42,280 bp in length with a G+C content of 51.2%, which was slightly lower than the average of the host genome (55.3%). According to the results of the VIRFAM analysis [57], the head–neck–tail structure genome organization of the OS31 phage belongs to *Siphoviridae* of type 1 (cluster 3). Surprisingly, electron micrographs performed on samples obtained by PEG precipitation only showed structures that are compatible with phage heads (~80–90 nm in diameter), but no attached tails and fibers were observed (Figure S3). We also noticed that the coverage of sequencing was not evenly distributed over the entire length of the genome (Figure S4). From the position of the 19,032 nucleotide (that presumably constitutes the phage packaging site), the number of reads gradually decrease downstream, which might suggest that the packaging of the OS31 genome is prematurely terminated. If that were the case, virion production would be significantly impaired, which might explain the lack of properly assembled viral particles in mitomycin-induced products.

In the *Serratia* sp. OS31 chromosome, the borders of the OS31 prophage sequence are defined by a flanking 23 bp direct repeat (TCGCACATGTCGCAGTTGATGCA), which is probably the attachment site. The phage insertion site was located in the putative host ferredoxin gene, belonging to a family of proteins containing two (4Fe-4S) clusters (pfam13183). In the course of comparative analysis, we discovered that numerous viruses were integrated into homologous genes (see Section 2.4.4.).

The OS31 genome was predicted to encode 60 putative ORFs, which share similarity at the protein level with other sequences in GenBank and 42 (70%) of them have assigned functions. The positions, sizes, and putative functions of those proteins are listed in Table S3. No gene encoding tRNA was detected in the genome.

The set of OS31 functional modules is similar to BZS1 and their arrangement is as follows—integration/excision, early transcriptional regulation, replication, host cell lysis, packaging, and capsid and tail assembly (Figure 2). Genes located upstream from position 8181 were oriented leftwards, whereas those positioned downstream were predominantly transcribed rightwards.

OS31 proteins associated with integration and excision seem to be unique. A putative product of the *phiOS31_p01* gene had a C-terminal catalytic domain of tyrosine-based site-specific recombinases (pfam00589). An Arm DNA-binding domain (pfam13356) was also found in this protein. The crucial role of Arm-binding domains in integrase recombination was shown for the lambda phages [58]. However, a BLASTP search against the NCBI viruses database (taxon: 10,239) revealed no homologs for PhiOS31_p01 (cutoff 1 × 10^−5^), the same as that for a putative excisionase (PhiOS31_p02) containing an HTH domain (pfam12728). Next to the pair of these ORFs, a putative 3′-5′ exoribonuclease-encoded gene was present (*phiOS31_p03*). Its protein product belonged to the DnaQ-like (or DEDD) exonuclease superfamily [59], and was similar to an exonuclease of the *Enterobacteria* phage mEp460 (GenBank acc. no. YP_007112100.1. sequence identity 64.48%) and counterparts of other *Enterobacteriaceae* phages. However, genes encoding these proteins by enterobacteria phages were not located in the vicinity of their integrase gene.

Putative OS31 gene regulatory circuitry, which includes genes for two repressors, namely CI and Cro, resembles that found in other well-characterized lambdoid phages. The predicted phage repressor CI (PhiOS31_p14) possesses both a S24 family peptidase domain and a DNA-binding HTH domain. PhiOS31_p15 contains a conserved domain of the Cro repressor family of proteins (pfam09048) and PhiOS31_p16 contains a domain of phage regulatory protein CII (pfam06892). All these proteins show high similarity to their counterparts, encoded by various lambdoid enterobacteria phages.

The DNA region following the lysogeny module comprising *phiOS31_p18-19* genes is predicted to encompass the replication module, as the encoded proteins are similar to the DNA replication proteins of *Klebsiella pneumoniae* phages, for example [60]. A putative protein product of the *phiOS31_p18* gene belongs to the DEAD-like helicases superfamily (pfam00270), and PhiOS31_p19 belongs to the Toprim superfamily (pfam13362), which suggests it to be a DNA primase. A putative product of the adjacent gene *phiOS31_p20* was identified as NinG (Rap, pfam05766), which presumably supports the replication process as an auxiliary component. Genetic analysis implicated the phage λ Rap endonuclease targets as recombinant joint molecules arising from phage λ Red-mediated genetic exchange [61], and can function as a Holliday junction resolvase [62]. PhiOS31_p20 shares 44% sequence identity with λ Rap endonuclease. Interestingly, in both λ as well as in genomes of the abovementioned *Klebsiella pneumoniae* phages, NinG (Rap)-like genes are not located in close proximity to replication genes.

Downstream of the replication module, there are two genes, namely, *phiOS31_p23* and *phiOS31_p24*, whose protein products showed no viral homologs. Within PhiOS31_p23, the GepA-like (COG3600) and DUF4065 (pfam03629) domains were identified on the basis of the HHpred searches (99.87 and 99.58% probabilities, respectively). GepA (standing for genetic element protein A) proteins, which carry the DUF4065 domain, were previously associated with the toxin–antitoxin (TA) loci and were related to proteolysis-promoting SocA antitoxins [63]. In this new type (VI) of TA system, the SocA antitoxin functions as a ClpXP protease adaptor for the SocB toxin, promoting degradation of the toxin and thereby abolishing its lethality [64]. PhiOS31_p23 shows high similarity with bacterial proteins annotated as the SocA family. On the other hand, the HHpred search of PhiOS31_p24 identified a hit to mRNA-degrading endonucleases (COG2337) of type II toxins of MazEF-like TA systems (98.94% probability) [65]. Furthermore, the sequence closely matching the *E. coli* consensus ribosome binding site (5′-AGGAGG-3′) is located immediately upstream of *phiOS31_p23*, and the stop codon of *phiOS31_p23* overlaps with the ATG of *phiOS31_p24* by a single base pair. This suggests transcriptional and translational coupling of the two open reading frames. Organization of TA pairs as a single operon co-transcribed from the same promoter is typical for various TA systems, e.g., the *mazE* and *mazF* genes of type II [66]. Although all these features allow the presumption that *phiOS31_p23* and *phiOS31_p24* form a new TA system, this hypothesis needs experimental validation.

Similar to BZS1, a putative type II m6A MTase was also identified in the OS31 genome. The protein product of the *phiOS31_p25* gene was similar to PhiARM81ld_p56 (58% identity) encoded by a linear plasmid-prophage PhiARM81ld of *Aeromonas* sp. ARM81. ARM81ld_p56 is an unusual adenine MTase, which has no sequence specificity and modifies adenine residues in most sequence contexts [33]. Substrate specificity of PhiOS31_p24 seems to also be relaxed, since the plasmid DNA of pET_PhiOS31_p25 isolated from an IPTG-induced *E. coli* ER2566 was partially resistant to cleavage with various restriction endonucleases (REases) sensitive to m^6^A in their recognition sequences, namely, HinfI, (GANTC), PfeI (GAWTC), SchI (GAGTC), Hin1II (CATG), Tru1I (TTAA), and TasI (AATT). Additionally, pET_PhiOS31_p25 isolated from an IPTG-induced *E. coli* ER2929 Dam^−^ strain was resistant to cleavage with MboI but was sensitive to DpnI. On the other hand, all abovementioned REases cleaved DNAs of pET_PhiOS31_p25 isolated from uninduced *E. coli* strains (controls) and produced the expected restriction pattern (Figure S1b). Results of this endonuclease protection assay indicate the presence of m^6^A in various sequence contexts of DNA isolated from cells expressing PhiOS31_p25, which suggests its extremely relaxed substrate specificity. Interestingly, homologs of PhiOS31_p25 were found by us in numerous viruses, e.g., *Enterobacteria* phage phiP27 (GenBank acc. no. NP_543076.1, 67% identity), *Morganella* phage IME1369_02 (ARM67989.1, 66% identity), and many *Escherichia* phages, which suggests that they are also non-specific DNA MTases. 

Deduced products of the host lysis cassette are formed by holin and lysozyme genes *phiOS31_p26-27*, respectively, and a pair of spanins (*phiOS31_p28-29*). Such a genetic arrangement is common among lambdoid phages [40], but no known virus encodes a full set of protein homologues to PhiOS31_p26-29.

Downstream of the lysis module, there is a packing module formed by genes *phiOS31_p36-37*. The PhiOS31_p36 protein belongs to the Terminase 4 superfamily (pfam05119), while the PhiOS31_p37 protein belongs to the Terminase 1 superfamily (pfam03354). This indicates that these proteins are small and large subunits of terminase, respectively. Both subunits revealed similarity (sequence identities of 83.36% and 88.96%, respectively) to their counterparts of *Klebsiella* ST13-OXA48 phages, namely, phi12.2 (GenBank acc. no. QBP27572.1, QBP27573.1), phi12.4 (QBQ71912.1, QBQ71911.1), phi17.2 (QBP28014.1, QBP28013.1), and also many *Streptococcus* phages. Putative OS31 terminase genes are preceded by *phiOS31_p34*. Products of this gene belong to the HNHc endonuclease superfamily (cd00085). It was widely reported that that phage genomes often encode proteins possessing an HNH motif near their terminase genes, suggesting a possible biological role in the stimulation of homologous recombination by nicking DNA, which further enhances gene conversion. PhiOS31_p34 is most similar to a protein of the *Aeromonas* phage AsXd-1 (sequence identity 75.45%) [67] and also to endonucleases of the previously mentioned *Klebsiella* phages (QBP27571.1, QBQ71913.1, and QBP28015.1).

Almost all putative products of the OS31 structural gene cluster are homologues to the HK97 structural proteins. HK97-fold lineage is one of four lineages of viral capsid proteins [68,69]. The structural gene cluster of OS31 covers similarly oriented ORFs from *phiOS31_p38* (predicted as portal protein) to *phiOS31_p57* (predicted as tail fiber). OS31 proteins that are presumably involved in head formation (portal, prohead protease, major capsid protein, PhiOS31_p38-40) show a higher degree of identity with HK97 (80←86%) than head–tail connectors (PhiOS31_p41-42, 61% and 44%, respectively) and tail proteins PhiOS31_p43-44 and p47-56 (43–57%). We did not found HK97 counterparts for only PhiOS31_p54-55 and PhiOS31_p57, which share similarity with other *Escherichia* phages, e.g., the Fraca (GenBank acc. no. VUF53278.1 and VUF53299.1, sequence identity 44% and 60%) and *Serratia* phage Serbin (QBQ72943.1, sequence identity 33%) proteins. PhiOS31_p54 contains an N-terminal sorting signal that favors translocation into the outer membrane, conforming to the Prosite consensus for prokaryotic lipoprotein lipid attachment sites (amino acids 1–16: MKKVLITALASFLLSGC; potential lipidation site, C17 is underlined) [70]. The *phiOS31_p54* location next to tail fiber genes and its size suggests that its product might be an analog of Cor proteins, e.g., of Rtp, T1, HK022, phi80, and N15 phages, which confer resistance to heteroimmune phage infection, by inactivating the receptors [71,72].

Interestingly, in the middle of the OS31 structural module there is a pair of oppositely oriented genes, namely, *phiOS31_p45* and *phiOS31_p46*, whose putative products belong to the Tra5 superfamily (COG2801) and InsE superfamily (COG2963), respectively, indicating that they form a mobile element. BLAST searching results from the ISfinder database revealed that *phiOS31_p45-46* encoded proteins are homologous to the transposases of insertion sequences (IS) belonging to the IS150 group of the IS3 family. Members of the IS3 family show a general structure, consisting of a single transposase gene, flanked by terminal inverted repeats, and the transposase gene contains two open reading frames, namely, *orfA* and *orfB* [73]. For the representatives of IS3, including the IS150 group, *orfA* and *orfB* partially overlap and the OrfAB transposase is produced by programmed −1 translational frameshifting between *orfA* and *orfB* [74]. This process takes place within the motif A_n_G that is present in the overlapping region [75]. Bioinformatics analysis revealed that the OS31 IS contains both *orfA* and *orfB*, which were *phiOS31_p46* and *phiOS31_p45*, respectively. The sequence AAAAAAG (coordinates 26,840–26,846), located directly upstream of the *phiOS31_p46*, might be responsible for such a frameshifting event to produce a 456 aa transframe protein, i.e., the transposase. We also identified 13 bp inverted repeats of TGAAGTGAACCCC, which seem to constitute the boundaries of this 1437 bp mobile element (named ISSspOS31). Moreover, 4 bp (TCCG) directly repeated sequences were found, presumably generated as the result of target site duplication upon IS insertion. It is worth noting that the GC content of ISSspOS31 was only 45.1%. Additionally, examination of ISSspOS31 OrfAB revealed the presence of an HTH domain within the N-terminal region and a distinctive DDE catalytic motif of the related integrases of retroviruses [76] in the C-terminal of a predicted OS31 transframe OrfAB (D298 D357 E393), and also the presence of a conserved lysine residue of six amino acids, downstream of the glutamate residue (K400). 

We identified IS elements similar to ISSspOS31 in several *Yersinia* genomes and usually in multiple copies, e.g., *Yersinia enterocolitica* subsp. enterocolitica 8081 (GenBank acc. no.AM286415.1, coordinates: 3544804-3546239; 4176235-4174800, 1791929-1793364, 72% identity). It must be emphasized that none of these IS copies were localized in a prophage sequence. In-depth bioinformatic analysis revealed that the vast majority of phage genomes (92%) did not contain any IS insertion [77]. It was suggested that phages were less tolerant to IS, i.e., they had a lower cargo capacity than other mobile genetic elements of horizontal transfer plasmids [77,78]. Given this, the presence of an IS element in the OS31 phage genome seemed to be exceptional.

We were also looking for IS elements similar to the ISSspOS31 in phage genomes. A similar genomic segment was found in the *Bacillus* phage vB_BtS_BMBtp14 (GenBank acc. no.KX190833.1, coordinates 57-601). BMBtp14 OrfB (ANT39961.1) and PhiOS31_p45 shared 54% sequence identity, while a putative OrfA PhiOS31_p46 and BMBtp14 OrfA ANT39962.1 showed 33% sequence identity. PhiOS31_p45 homologs were also identified in several *Streptococcus* phages (e.g., Javan18), *Paenibacillus* phage Xenia, and *Shigella* phages (e.g., SfIV and Sf6). Surprisingly, we also found nucleotide similarity between ISSspOS31 and an *E. coli*-derived transposable element in the *Macacine betaherpesvirus* 3 (Rhesus cytomegalovirus) genome (coordinates 174918-175257, 66% identity). Moreover, two proteins encoded by the latter one (annotated as IS150 transposase A and B; AUI39789.1 and AUI39788, respectively) showed similarity with PhiOS31_p45 and PhiOS31_p46 (55% and 30% identity, respectively).

Downstream of a cluster of tail protein encoding genes, in the opposite orientation, there are *phiOS31_p59* and *phiOS31_p60* genes, of which the latter show SOS-related predicted functions, as its putative product is similar to DinI-like proteins. The DinI of *E. coli* was shown to modulate the bacterial SOS response by inhibiting activated RecA-mediated LexA protein autocleavage. Interestingly, the sequence 5′-TACTGGCTATATATACAGCATA-3′, which lies 14 bp upstream of the *phiOS31_p60* gene, is an almost perfect match to the consensus of an *E. coli* LexA binding site (5′-taCTGtatatatataCAGta-3′, where uppercase nucleotides are universally present and lowercase nucleotides are not absolutely conserved). DinI-like genes are found in numerous temperate phage genomes, e.g., *Klebsiella* PhiKO2, *E. coli* 186, and prophages, e.g., Gifsy-1, Gifsy-2, and Fels-2 of *Salmonella* LT2 [79,80]. It was shown that the PhiKO2 *din*-like gene is regulated by the host’s LexA repressor [79]. PhiOS31_p60 share 60.3% identity with the protein product of this gene. The PhiKO2 *din*-like gene, similar to *phiOS31_60*, is also divergently oriented, relative to the tail fiber gene. Interestingly, *phiOS31_p60* stop codon overlaps with ATG of the neighboring *phiOS31_p59*, by a single base-pair, suggesting transcriptional and translational coupling of the two open reading frames.

A protein product of the *phiOS31_p59* gene showed no viral homologs, but similar hypothetical proteins were identified in several *Serratia* spp. genomes, e.g., AGQ32447.1 of *S. liquefaciens* ATCC 27592 (92% identity). It is worth emphasizing that also in this case there was a *dinI*-encoding gene in the vicinity, and both genes were located in a prophage sequence and in opposite directions to other genes. The organization of this gene cluster suggests cooperation between genes.

### 2.4. Comparative Genomic Analyses

#### 2.4.1. vB_SspM_BZS1 vs. vB_SspS_OS31

One segment of BZS1 genome (coordinates 42303-42963) shares significant nucleotide similarity with the OS31 genome (86% sequence identity, 456 bp, coordinates 16,802–17,258) (Figure 2). Putative spanin-like proteins encoded in these regions, i.e., PhiBZS1_p68 and PhiOS31_p28, and PhiBZS1_p69 and PhiOS31_p29, share 77.1% and 74% sequence identity, respectively. Additionally, two other pairs of BZS1 and OS31 proteins are also homologous—PhiBZS1_p35 and PhiOS31_p06 (HigB-like proteins, 37.1% sequence identity) and PhiBZS1_p48 and PhiOS31_p07 (ParB-like proteins, 35.5% sequence identity), respectively. It is worth mentioning that PhiBZS1_p27 and PhiOS31_p57 also show similarity, but this is limited to only a relatively short segment (~125 aa, 33% sequence identity) of the N-terminal part of these large >750 aa tail fiber proteins (Table S4). 

#### 2.4.2. vB_SspM_BZS1 and vB_SspS_OS31 vs. other *Serratia* Phages

**Figure 3:**
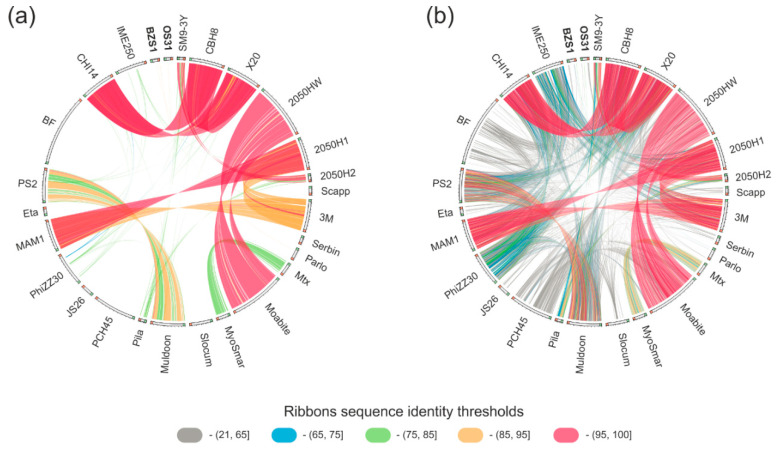
Comparison of BZS1 and OS31 with other *Serratia* phages. Similarities were based on nucleotide (**a**) and protein (**b**) all against all BLAST searches. Similarities are reflected by the ribbons connecting ideograms. Their colors reflect the scale of similarity between phage genome parts (**a**) or phage-encoded proteins (**b**), as indicated in the legend. The anchors of ribbons correspond to protein-encoding genes location. Green and orange rectangles at the ends of ideograms reflect the beginning and end of phage genome.

Currently, there are 25 *Serratia* phage annotated genome sequences available in the NCBI Viral RefSeq database (Table S1). The BZS1 genome only shares limited nucleotide sequence identity (coordinates: 28,219-28,599) with podovirus Parlo (GenBank MK618715.1; 89% sequence identity within a 380 bp region, coordinates 16,844-17,224), while OS31 shows no discernible DNA sequence similarity to any of *Serratia* viruses currently available in the NCBI viral database (Figure 3a). Therefore, the BZS1 and OS31 genomes differ significantly from the previously reported *Serratia* phage genomes at the nucleotide level.

BLASTP analyses (Table S4, Figure 3b) showed that the BZS1 proteome has 3 homologs with the abovementioned Parlo, namely, a putative chaperone_DnaJ (PhiBZS1_p45) and two hypothetical proteins (PhiBZS1_p38 and PhiBZS1_p49), as well as four with siphovirus JS26 (which is the large subunit of the terminase (PhiBZS1_p02)), and structural proteins, such as portal protein (PhiBZS1_p04), head-tail preconnector protease (PhiBZS1_p05), and a major capsid protein (PhiBZS1_p08). Interestingly, PhiBZS1_48 showed 55.5% sequence identity with the ParB homolog of Eta (GenBank acc. no. YP_008130295.1), the previously mentioned peculiar phage which forms unstable lysogens and whose genome featured no identification of integrase or repressor encoding genes. It was suggested that the Eta prophage is maintained as a plasmid-type element and is segregated using a combination of the phage ParB homolog (centromere-binding protein) and host factors [7]. The ParB-like protein of Eta also showed similarity but was lower (31.5% sequence identity) with PhiOS31_07. Additionally, PhiOS31_p18 and _p27 predicted the DEAD-like helicase and lysozyme, respectively, have homologous counterparts in Eta. Four putative tail structural proteins of OS31 (PhiOS31_p52-53 and _p55-56) share similarities with the siphovirus Scapp [81] (sequence identity between 38–43%) and one (PhiOS31_p57, 33% sequence identity) with the siphovirus Serbin [82] (Table S4, Figure 3b). The last one is a putative tail fiber protein. It is worth mentioning that contrary to PhiBZS1_p27 (see above), a region of similarity covers the entire protein sequences.

In summary, BLASTP similarity searches performed for the BZS1 and OS31 protein sequences and proteins encoded by 25 other *Serratia* specific phages mostly revealed a low level of amino acid sequence identity between a few (1 to 4) proteins, which suggests the lack of a significant phylogenetic relationship between both of them and between BZS1, OS31, and the other analyzed *Serratia* phages. In other words, BZS1 and OS31 are clearly remote from all other known *Serratia* phages. The analyses also showed the high diversity of *Serratia* phages in general (Figure 3). Six distinct groups of phages showed high or significant reciprocal similarity, but they gathered only two or three members, namely, (i) CHI14, CBH8, and X20, (ii) 2050HW and Moabite, (iii) 3M, 2050H1 and MAM1, (iv) SM9-3Y and 2050H2, (v) PS2 and Muldoon, (vi) and MTx and MyoSmar. The other 13 phages showed either weak (below 65% of amino acid sequence identity) or no similarity to other *Serratia* viruses. Together, this resulted in 19 mutually dissimilar groups of *Serratia* phages (21 including BZS1 and OS31).

#### 2.4.3. vB_SspM_BZS1 and vB_SspS_OS31 vs. All Known Phages

**Figure 4:**
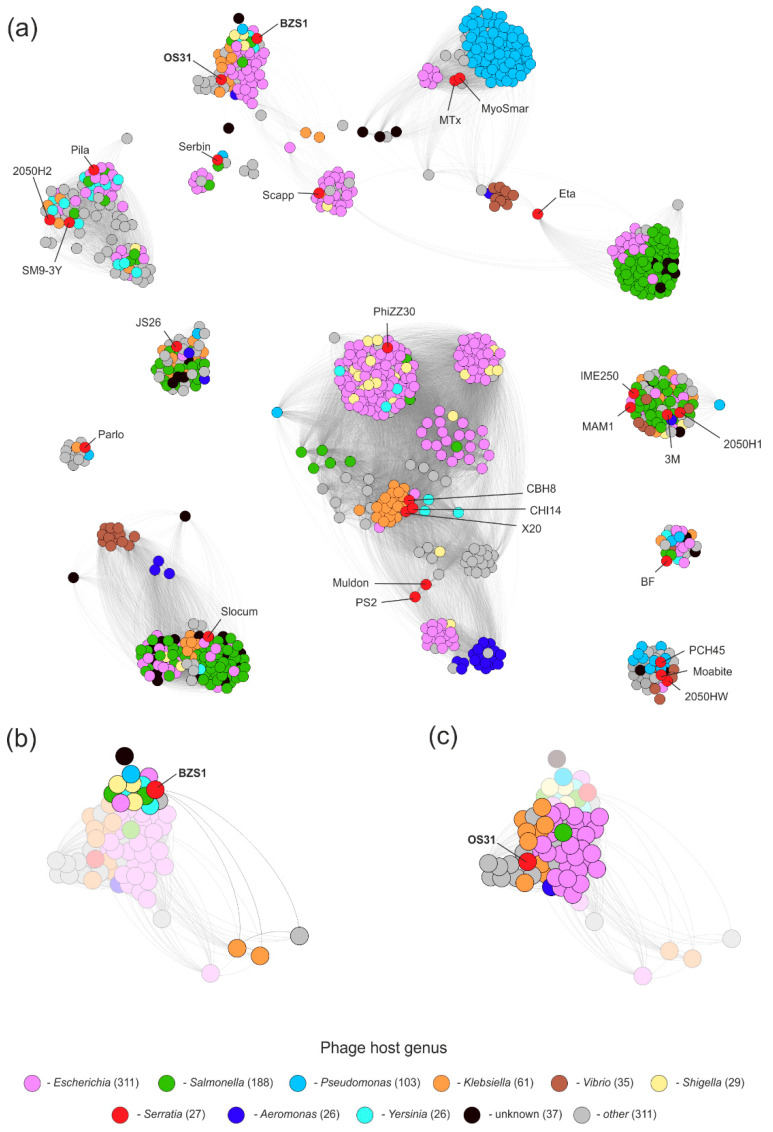
Protein-based phage similarity network. Phage genomes from NCBI were compared with BZS1 and OS31 phages with the application of vConTACT v0.93. On the network, only phages exhibiting similarity to *Serratia* phages were considered to yield a network of 1039 nodes – panel (**a**). In particular, phages showing similarity to BZS1 and OS31 phages were highlighted on panel (**b**,**c**), respectively. Node colors were used to indicate phage hosts according to the legend.

**Figure 5:**
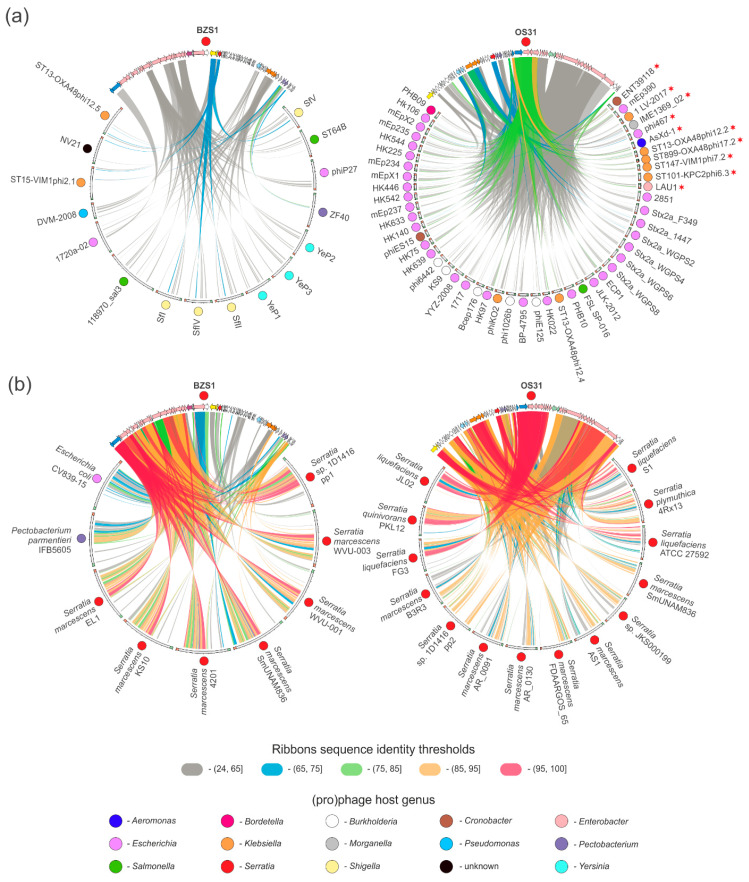
The comparison of BZS1 and OS31 phages with similar (pro)phages. The comparison was based on the comparison of BZS1- and OS31-encoded proteins against (**a**) proteins encoded by phages they were connected to in the network analysis and (**b**) prophages from bacterial genomes. The threshold of sequence identity shown with ribbons and (pro)phages’ hosts genus were labeled as shown on the legend. The anchors of ribbons correspond to protein-encoding genes location. Green and orange rectangles at the ends of ideograms reflect the beginning and end of phage genome. Red stars were used to indicate phages that clustered with OS31 phage in vConTACT analysis.

As the comparison of BZS1 and OS31 viruses with other *Serratia* phages did not reveal any close relatives, these were compared to all phages present in the GenBank database (as of 28 April 2020) via the application of vConTACT v0.93. The resulting proteome-based phage similarity network, limited to phages exhibiting a relationship with either BZS1, OS31, or other *Serratia* phages, was composed of 1039 phages in total (Figure 4a). Each node of the network, representing a single phage, was deployed on the basis of the degree of vConTACT’s proteome’s similarity with the other one, i.e., higher the overall similarity, the closer were the two nodes deployed with the application of ForceAtlas 2 layout in Gephi. Within the network, the *Serratia* phages were spread across 15 different viral clusters and 6 of them were considered to be outliers, according to vConTACT, confirming the previous observations. Phages BZS1 and OS31, although grouped in a single cluster of phages encoding homologous proteins, shared distinct similarity to phages between them (Figure 4b,c). This was also reflected in different clusters assigned by vConTACT (Figure S5). BZS1 was in fact classified as one of the outliers, as its similarity to 15 other phages was based only on single proteins, which further confirmed its uniqueness (Figure 5a, left panel, Figure S5a, Table S5). In contrast, OS31 created a viral cluster with 14 other phages, infecting *Enterobacteriaceae* (in total 11, i.e., *Klebsiella* (8)*, Enterobacter* (2), *Escherichia* (1), and *Cronobacter* (1)), as well as *Morganellaceae* (2, i.e., *Morganella* and *Proteus*), and *Aeromonadaceae* (1, *Aeromonas*) (Figure S5b, Table S5). The exploration of similarities revealed that those were observed for 70% proteins encoded by OS31 (Figure 5a, right panel), although the range of sequence identity was mostly between 24–65%, with the exception of two proteins yielding higher identities, i.e., a small terminase subunit (PhiOS31_p36, 88%) and a major capsid protein (PhiOS31_p40, 84%). Nevertheless, the similarity between OS31 and individual phages of this group was based at most on 20 proteins, which are mainly structural (Table S6).

The analysis also showed the overall diversity of known *Serratia* phages that spread across different clusters of viruses. Barely a few of the previously mentioned highly similar phages clustered together. This indicates the high diversity of phages infecting *Serratia* and potentially a low saturation of the diversity discovered here. It is worth mentioning that an interesting location of the Eta phage, which is located between the two clusters of mostly *Vibrio* and *Salmonella/Escherichia* phages, acts as a bridge between the two clusters (Figure S6). Detailed insight into protein similarities responsible for its location revealed its hybrid/mosaic genome, in which, one part—the encoding proteins—was potentially involved in genome replication, showing similarity to the *Vibrio* cluster, while the other part, in which proteins involved in DNA packaging and virion assembly are encoded, was similar to *Enterobacteriaceae* phages.

#### 2.4.4. Prophages in *Serratia* Genomes Similar to vB_SspM_BZS1 and vB_SspS_OS31

The global comparative analysis of BZS1 and OS31 phages demonstrated uniqueness of BZS1 and limited resemblance of OS31 to known viruses. Since both these phages are temperate, we asked whether similar prophage sequences are present in bacterial genomes. Therefore, we searched bacterial genomes for the presence of prophages that could be closer relatives to analyzed phages. With the application of nucleotide searches against the non-redundant nucleotide NCBI database, we selected bacterial chromosomes that yielded significant similarity to BZS1 and OS31 genomes (threshold minimal query coverage 24%). As a result, we identified 9 and 14 prophage regions exhibiting similarity to BZS1 and OS31 phages, respectively. These regions were manually inspected, which revealed that each of them were potentially a complete prophage genome (Table S7). Those prophages were compared with BZS1- and OS31-encoded proteins to reflect the scale of their resemblance (Figure 5b). Overall, the observed similarities between BZS1 and OS31 and the indicated prophages were significantly higher than their similarity to phages from the network analysis (Figure 5a). In the case of BZS1, it showed the highest similarity to prophages identified in *Serratia* spp. genomes, although the two were also identified in *Escherichia coli* CV839-15 and *Pectobacterium parmentieri* IFB5605. It is also worth noting that high similarity was observed only for proteins involved in DNA packaging, structure, and capsid assembly, except PhiBZS1_p24 (predicted as peptidoglycan/LPS O-acyltransferase). However, it should be stressed that 6 of these prophages (5 of *Serratia* spp. 1D1416, 4201, KS10, SmUNAM836, EL-1, and one of *E. coli* CV839-15) encode putative O-acyltransferase but are not homologous to PhiBZS1_p24. Similarly, protein products of genes located in the other part of the BZS1 genome (comprising, for example, replication and lysogeny modules) show low or no similarity to proteins encoded by prophages, as shown in Figure 5b (left panel). On the other hand, for OS31, the analysis revealed the presence of 14 *Serratia* spp. prophages encoding protein homologs for 88% of OS31 proteins. However, only the *Serratia plymuthica* 4Rx13 prophage showed very high similarity (over 85% protein sequence identity) to OS31, across its whole genome (Figure 5b, right panel). The few proteins that remained unique for OS31 were either hypothetical (e.g., PhiOS31_p21-24, including a presumed TA system) or IS proteins.

It is also worth noting that among all prophages identified by comparative analyses, two were carried by the same strain, namely, *Serratia* sp. 1D1416. The pp1 and pp2 showed resemblance to the BZS1 and OS31 phages, respectively. Moreover, a prophage carried by *Serratia marcescens* SmUNAM836 seemed to have a hybrid (chimeric) genome similar to both the BZS1 and OS31 phages (Figure S7), combining the structural genes (although without homologous acetyltransferase) from the first one and the non-structural genes (including integrase, lysogeny control, replication proteins, DNA methyltransferase, holin, and lysozyme) from the latter one.

### 2.5. Functional Characterization of the vB_SspM_BZS1 and vB_SspS_OS31 Phages

#### 2.5.1. Host Range

The majority of bacteriophages are capable of infecting a narrow range of bacteria that are closely related [83]. However, some polyvalent phages were reported, predominantly among viruses of Enterobacteria [84,85,86,87] and staphylococci [88]. These polyvalent phages can infect strains from either different genera or species. For example, *Serratia* phage MAM1 is a broad host range virus that infects *Serratia* spp. and *Kluyvera* spp. [89], and Phi 0T8 was shown to infect *Serratia* sp. ATCC 39006 and *Pantoea agglomerans* [90].

The host range of phages BZS1 and OS31 was tested on 18 bacterial strains (including *Cronobacter* spp., *Yersinia enterocolitica*, *Salmonella* spp., *Escherichia coli*, and environmental and clinical strains of *Serratia* spp.) via spot testing of diluted phage lysates. The bacterial strains used in this assay are listed in Table S8. *Serratia* spp. isolates from the Zloty Stok mine (including the original host strain OS31) were susceptible to infection by BZS1. While no plaques were obtained with any other strains tested, including a clinical strain of *Serratia* sp. This might suggest that BZS1 has a narrow host range, possibly confined to the *Serratia* spp. strains inhabiting the rock biofilms of the Zloty Stok mine. In contrast, no lytic growth of OS31 could be observed on any of the tested bacterial strains. In the case of environmental isolates of *Serratia* spp. from the Zloty Stok biofilms, it could not be excluded that these were immune to OS31 infection as they carry the same or related prophage. Another explanation of the OS31 inability to propagate in any of the strains tested might be that most of the OS31 particles seem to be defective, due to the lack of a tail and fibers (see Section 2.3), which is necessary for host recognition by *Caudovirales*.

Since none of the tested bacterial strains were sensitive to OS31, further physiological analyses as a one-step growth curve, adsorption rate, and phage stability, were performed only for BZS1.

#### 2.5.2. Adsorption Assay and One-Step Growth Curve of vB_SspM_BZS1

The rate of phage adsorption to host cells, the number of released phages after lysis, and the burst size, are basic biological properties of any virus, and are values characteristic of a phage–host pair.

**Figure 6:**
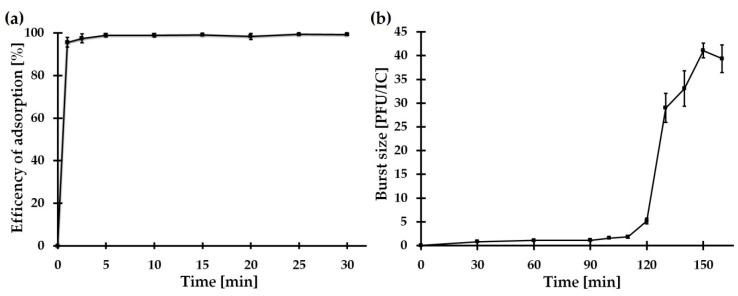
Phage BZS1 development. Adsorption of phage particles to the bacterial cells assessed after 1, 2.5, 5, 10, 15, and 30 min (**a**). One-step growth curve (**b**). Each data point represents the mean of three independent experiments.

A phage adsorption assay revealed that more than 99% of the BZS1 phages were rapidly adsorbed onto the *Serratia* sp. OS31 host cell within 5 min (Figure 6a). A one-step growth curve experiment showed that BZS1 had a latent period of 120 ± 5 min and a rise period of 150 ± 5 min (Figure 6b). In comparison to other phages that infect *Serratia* spp., BZS1 has relatively long latency and rise periods. For example, vB_SmaA_2050H1 had a latent period of 80 min and a rise period of 50 min, vB_SmaM_2050HW showed a latent period of 40 min and a rise period of 60 min [91], and CBH8 and MAM1 had a latent period of 20 and 25 min and a rise period of about 35 and 40 min, respectively [89,92]. BZS1 exhibited an average burst size (40 ± 2 phages per infected cell) compared to the abovementioned *Serratia* phages, with the following plaque forming units (PFU) per infected cell: vB_SmaA_2050H1, 966; vB_SmaM_2050HW, 113; MAM1, 300; CBH8, 22 [89,91,92].

#### 2.5.3. vB_SspM_BZS1 Stability under Various Conditions

**Figure 7:**
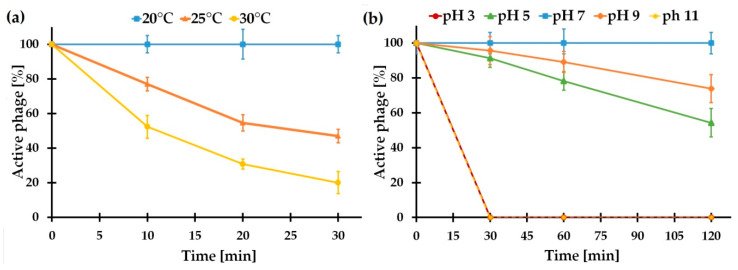
Phage BZS1 stability under various conditions. Effect of pH on the phage activity (**a**). Effect of temperature on the phage activity (**b**).

The BZS1 phage showed almost 100% stability under pH values ranging from 7.0 to 9.0, while the titers of the phage decreased with increasing acidity or basicity (Figure 7a). Moreover, BZS1 was very sensitive to temperature. The phage titer started to reduce at 25 °C (Figure 7b). Higher temperatures (over 40 °C) resulted in an immediate loss of activity. Although, the temperature stability of BZS1 seemed to not be high, it was adequate for the environment it originated from. The end and middle sections of Gertruda Adit of Zloty Stok mine (the place of biofilm sampling) were characterized by a stable air temperature of 10.4–11.1 °C and stable water temperature of 10–12 °C, throughout the year [93]. The pH of the Zloty Stok mine water was neutral (7.4–8.1) [93], thus, corresponding to the range tolerated by the virus.

## 3. Materials and Methods

### 3.1. Bacterial Strains, Plasmids, and Culture Conditions

The bacterial host of investigated phages *Serratia* sp. OS31 was isolated from rock biofilms of the Zloty Stok gold and arsenic mine (SW Poland) using the methods described previously [93] and was kindly provided by Lukasz Drewniak. The strain was grown under aerobic conditions in lysogeny broth (LB) or R2A media [94] at 20 °C. Using the same methodology [93], we isolated two other *Serratia* spp. strains (BZSmr3 and BZSmr6) from biofilms of the walls of the Gertruda Adit in the Zloty Stok in April 2018. They were used in host range testing assay. Bacteria used in host range testing (Table S8) were grown under aerobic conditions in LB at 30 °C, except *Yersinia eneterolitica* 2/O:9 (at room temperature, RT) and environmental *Serratia* spp. strains from Zloty Stok (at 20 °C).

The following *Escherichia coli* strains were used in this study: TOP10F’ (Invitrogen, Waltham, MA, USA), ER2566 (New England BioLabs, Ipswich, MA, USA), and ER2929 Dam^−^ strain lysogenized with λDE3 element, which carried the gene for T7 RNA polymerase under control of the lacUV5 promoter [95]. These were cultured under standard conditions in LB medium at 37 °C. When required, the media were supplemented with kanamycin at 50 μg·mL^−1^, and ampicillin at 100 μg·mL^−1^. Plasmids pUC19 (Thermo Fisher Scientific, Waltham, MA, USA) and pET30a (Invitrogen) were used as cloning or expression vectors.

### 3.2. Isolation of the vB_SspM_BZS1 Phage

Sampling site and conditions in this environment was described elsewhere [93]. Rock biofilm samples were collected from Zloty Stok mine in April 2018. The samples were centrifuged at 12,000× *g* for 10 min to remove the solid impurities, the supernatants were percolated through a 3-μm pore-size, low-protein-binding membrane filter (MerckMillipore, Darmstadt, Germany), then filtered through a 0.22 μm pore-size membrane filter (MerckMillipore), to remove the bacterial cells and sample debris and to obtain crude phage filtrates. Subsequently, a spot test was performed to determine the presence of phages. Briefly, 0.2 mL of the bacterial host *Serratia* sp. OS31 culture and 3 mL of molten top LB agar (0.7% agar) were mixed, then spread on solid LB medium to create a bacterial lawn. Ten microliters of the crude phage filtrate was spotted on bacterial lawn. The plates were incubated at 20 °C for 24 h, and then were inspected for lysis zones. Plaques were punched out using a sterile pipette tip and put into 1 mL of saline magnesium (SM) buffer [100 mM NaCl, 50 mM of Tris Cl (pH 7.5), 8 mM of MgSO_4_·7H_2_O] in 2 mL Eppendorf tubes. The vials were kept at RT to allow the phages to diffuse from the agar into the buffer, and then centrifuged at 12,000× *g* for 10 min. The supernatant was filtrated with a 0.22 μm pore-size Millipore Millex-GV Syringe Filter Unit (MerckMillipore). Bacteriophages were purified from single plaque isolates using the soft agar overlay method. The purification process was repeated three consecutive times. The purified phages were propagated using *Serratia* sp. OS31 host bacteria.

To obtain high titer lysates (10^10^ PFU·mL^−1^), the plates showing near confluent lysis were selected and soaked with 4 mL of SM buffer, and then incubated at 4 °C for 2 h. The liquid was removed, filtered, and stored at 4 °C, until required. Phage titers were determined using plaque assay, and 10-fold serial dilutions. Phage DNA was isolated by phenol–chloroform extraction and isopropanol precipitation [94], and analyzed by 0.7% agarose gel electrophoresis.

### 3.3. Isolation of the Lysogenized Strain of Serratia sp. OS31

BZS1 phage was plated with its *Serratia* sp. OS31 host using the LB agar overlay method [96]. After >24 h of incubation, colonies in the lysis zones were streaked on to a fresh LB plate, and purified after three consecutive single colony isolations. The lysogenized strain was named *Serratia* sp. OS31L_BZS1_.

### 3.4. Induction of vB_SspS_OS31 Prophage

vB_SspS_OS31 prophage of *Serratia* sp. OS31 was induced by mitomycin C (MilliporeSigma, Darmstadt, Germany). The bacterial culture was grown to optical density at 600 nm (OD600) of 0.4. The culture was then treated with mitomycin C (500 ng·mL^−1^), and its growth (with shaking) was continued for 6 h. The phage particles were precipitated from the lysate through the standard methods [94]. Phage DNA was isolated as in Section 3.2.

### 3.5. DNA Sequencing

Next generation sequencing of phage and bacterial DNAs was performed by DNA Sequencing and Oligonucleotide Synthesis Laboratory IBB PAN (Warsaw, Poland) and Biobank Lab, University of Lodz (Lodz, Poland), respectively. Genomic DNA of *Serratia* sp. OS31 (extracted using a kit Genomic Mini, A&A Biotechnology, Gdansk, Poland) from bacterial cells harvested by centrifugation of an overnight culture carried out in LB medium, was used to prepare Illumina paired-end libraries with the TruePrep DNA Library Preparation Kit protocol (Vazyme Biotech, Nanjing, China). Whole-genome shotgun sequencing was performed on the Illumina MiSeq instrument (Illumina, San Diego, CA, USA), at a read length of 2 × 250 bp. Phage DNA sequencing was also performed with the application of an Illumina MiSeq instrument in the paired-end mode, using a v3 chemistry kit (Illumina). Raw reads were processed with fastp v0.19.5, with a sliding window of size 10 bp, to remove low quality (<Q15) at 3′ ends, as well as polyX tracks and reads shorter than 150 bp [97]. Filtered reads were afterwards carefully assembled with SPAdes v3.11.1 [97]. Filtered raw reads were also used in PhageTerm [17] to determine the BZS1 termini and the possible packaging mechanism.

### 3.6. Genome Annotation

The annotation of analyzed phage genomes was initially conducted with the application of RASTtk [98] in phage mode, on the PATRIC website [99]. This was further manually verified and improved in Artemis [100] and Clone Manager 9 (Sci-Ed) software. The assignment of predicted protein functions was then verified and based on homology searches performed with (i) BLAST programs against the NR database, including domain searches with CD-Search and the PRIAM database [101,102,103], (ii) HHpred server using HHpred or HMMER tools against the PDB_mmCIF70_11_Oct, SCOPe70_2.07, COG_KOG_v.1.0, Pfam-Av33.1, and NCBI_Conserved_Domains (CD)_v3.16 or the nr50_1_Oct databases [104], as well as the Prosite server http://www.expasy.org/prosite [105]. The transmembrane helices were identified with the help of TMpred [106] and the TMHMM server v. 2.0 http://www.cbs.dtu.dk/services/TMHMM/. Putative tRNA genes were identified using the tRNAScan-SE [107] and ARAGORN programs [108]. A phage family search was carried out using VIRFAM [57].

### 3.7. Comparative Genomics

All performed comparative analyses were either BLAST- or DIAMOND-based. Nucleotide and protein BLAST programs were used to seek homology between BZS1 and OS31 phages, and other sequences in NCBI databases, i.e., NT, NR, GenBank, or RefSeq.

In particular, comparison of the BZS1 and OS31 phages was based on application of tBLASTx (thresholds: e-value 1 × 10^−10^ and minimum 50 nt alignment length) in EasyFig v2.2.3 [109]. The comparison of phages visualized with Circoletto [110] and Circos [111] (Figure 3, Figure 5, Figures S6 and S7) were based on nucleotide (with e-value ≤ 1 × 10^−100^) or protein (e-value ≤ 1 × 10^−15^ and query coverage per HSP ≥ 75%) BLAST searches. Prophages similar to BZS1 or OS31 phages were identified by nucleotide BLAST searching their whole genome sequences against the NT database, with the following threshold minimum query coverage of 24%. Selected hits were further manually examined in Artemis or Clone Manager to determine the ends of predicted prophage regions.

The global comparison of all phages deposited in NCBI Viral RefSeq database was conducted with the use of vConTACT v0.93 and the ProcaryoticViralRefSeq85-Merged database, applying DIAMOND v0.9.24 for protein clustering and ClusterOne v1.0 for protein and viral cluster analyses [112,113,114]. The resulting network was visualized in Gephi v0.92 using ForceAtlas 2 and Noverlap layouts [115,116]. Information about phage hosts was based on the content of parsed GenBank files, analyzing sequence description or host and lab host source qualifiers.

### 3.8. Verification of Attachment Sites of vB_SspM_BZS1 Phage 

The probable *att* sites of BZS1 were verified in vivo using PCR amplifications and subsequent sequencing. PCR primers were designed to amplify the left and right integration flanking regions (Table S9). The PCR products were cloned into pUC19 plasmid vector (Thermo Fisher Scientific). Plasmid DNA isolation was done using the Plasmid Miniprep DNA Purification Kit (EURx, Gdansk, Poland). 

### 3.9. Cloning, Overexpression, Purification, and Testing the Activity of DNA MTases

The DNAs encoding PhiBZS1_p32 and PhiOS31_p25 were amplified by PCR, using primers (Table S9) designed to incorporate NdeI and XhoI sites at the 5′ and 3′ ends of these genes, respectively. The amplified DNA fragments were cleaved with NdeI and XhoI and cloned into the NdeI/XhoI-predigested pET30a, creating pET_PhiBZS1_p32 and pET_PhiOS31_p25, respectively. Protein expression and restriction enzyme digestion protection assay were carried out, as previously described [117].

### 3.10. Transmission Electron Microscopy 

Photomicrographs were taken using a Tecnai T12 BioTwin electron microscope (FEI, Hillsboro, OR, USA) at ×49,000 magnification. A total of 2 μL samples of phage solutions were absorbed onto carbon-coated 400 copper mesh grids for 1 min, then excess of the solution was blotted away, specimens were washed 3 times in distilled water, stained with 1% uranyl acetate for 1 min and air-dried. The visualization of the phages was performed at the Core Facility of International Institute of Molecular and Cell Biology (IIMCB, Warsaw, Poland).

### 3.11. SDS-PAGE and Mass Spectrometry Protein Analysis

SDS-polyacrylamide gel electrophoresis (PAGE) was conducted as described previously [118]. The phage suspensions were heated with SDS-PAGE loading buffer (100 mM Tris-HCl [pH 6.8], 200 mM 2-mercaptoethanol, 4% sodium dodecyl sulfate, 20% glycerol, and 0.2% bromophenol blue) and loaded on one-dimensional 12% (*w/v*) SDS gel. After separation by electrophoresis, the gel was stained using Coomassie brilliant blue R-250 (Bio-Rad) and the protein bands were excised. Mass spectrometry analysis were performed in the Mass Spectrometry Laboratory, Institute of Biochemistry and Biophysics, Polish Academy of Sciences (IBB PAS) (Warsaw, Poland).

### 3.12. Adsorption Kinetics

The adsorption assay was performed as described previously [119]. BZS1 was added to *Serratia* sp. OS31 culture (multiplicity of infection, MOI = 0.01) and incubated without shaking at 20 °C. After 1, 2.5, 5, 10, 15, 20, 25, and 30 min, 100 µL samples were collected, and the phage-adsorbed cells were removed by centrifugation (5000× *g*, 1 min). The titer of unabsorbed phages was determined by double-layer plate titration. The number of adsorbed phages was determined, based on the ratio between the initial titer and test titers of unabsorbed phages. The data were obtained from three independent experiments.

### 3.13. One-Step Growth Curve 

For a one-step growth analysis, an overnight *Serratia* sp. OS31 culture was used to inoculate fresh LB medium. After reaching OD_600_ = 0.5, the culture was infected with the BZS1 phage at an MOI of 0.001 and incubated without shaking at 20 °C for 5 min. Next, the suspension of infected bacteria was diluted 10 times in fresh medium and incubated at 20 °C, with shaking (150 rpm). At appropriate time points, the numbers of PFU were determined by serial dilution and standard plaque assay. The number of infection centers (ICs) and the burst size presented as PFU/IC were calculated, as described previously [120]. The data were obtained from three independent experiments.

### 3.14. Thermal and pH Stability

For pH stability, the phage lysate (10^10^ PFU/mL) was diluted 100 times in LB broth, adjusted with NaOH or HCl, to yield various pH: 3, 5, 9, and 11. After 30, 60, or 120 min at RT, the numbers of PFU were determined by serial dilution and standard plaque assay. The control sample was incubated at pH 7.

For thermal stability, the phage lysate (10^10^ PFU/mL) was diluted 100 times in LB broth and incubated at 25, 30, 40, and 50 °C. After 10, 20, or 30 min, the numbers of PFU were determined by serial dilution and plaque assay. Control sample was incubated at 20 °C.

### 3.15. Determination of the Phage Host Range by Spot Testing

The phage host range was determined by a spot test assay on the potential host lawn plated onto LB agar plates. A total of 10 µL of the phage suspension (10^10^ PFU/mL) was added to the potential host lawn and then incubated at a suitable temperature. The plates were examined for the presence of bacterial lysis for 48 h.

### 3.16. Nucleotide Sequence Accession Numbers

The complete nucleotide sequence of the vB_SspM_BZS1 and vB_SspS_OS31 genomes could be accessed under the GenBank accession no: MT843275 and MT843276, respectively. The Whole Genome Shotgun project of *Serratia* sp. OS31 was deposited at DDBJ/ENA/GenBank under the accession JACJFQ000000000. The version described in this paper is version JACJFQ010000000. The 16S rDNA sequences of environmental *Serratia* spp. determined in this study were deposited in GenBank under the accession no. MT815621 and MT815622.

## 4. Conclusions

In this study, we described the identification of two novel *Serratia* temperate phages—myovirus vB_SspM_BZS1, forming turbid plaques on *Serratia* sp. OS31 (which can be stably lysogenized by this phage), as well as mitomycin C-inducible siphovirus vB_SspS_OS31, which exists as a prophage of *Serratia* sp. OS31. To the best of our knowledge, these are the first classical temperate *Serratia* phages that were thoroughly analyzed.

We showed that BZS1, like many other phages, has a narrow host range, possibly limited to *Serratia* spp., inhabiting rock biofilms of the Zloty Stok mine. Despite a quick adsorption, BZS1 has relatively long latency and rise periods, followed by a medium burst size, compared to other *Serratia* phages. BZS1 particles were stable in a pH ranging from 7.0 to 9.0, but sensitive to temperatures higher than 25 °C. Nonetheless, these properties seemed to be adequate to environmental conditions in the Zloty Stok mine.

In the case of the OS31 prophage, upon induction, it did not seem to be able to produce fully functional virions. In TEM analysis, OS31 particles appeared to be incomplete, as they were devoid of tails, even though the OS31 genome encodes information for these structures. Since in the NGS data we see a gradient of decreasing sequence coverage, starting from hypothetical packaging initiation region, we supposed that virion production was impaired due to the premature disruption of the packaging of the OS31 genome into the capsid. It could not be ruled out that OS31 is already undergoing degenerative changes towards a defective prophage. If such a hypothesis is correct, then OS31 might serve as an excellent model for studies on the evolution of temperate phages.

Genomes and proteomes of both BZS1 and OS31 are dissimilar to the known *Serratia* phages and show marginal (BZS1) or low (OS31) similarity to other viruses in public databases. Global comparative analysis of their proteomes confirmed the uniqueness of BZS1. Nevertheless, we discovered several putative prophage sequences in various *Serratia* (and also *Escherichia* and *Pectobacterium*) genomes, which showed significant similarity to BZS1 or OS31 or both (i.e., a chimeric phage). The global comparative analyses also showed the large diversity of *Serratia* phages in general, since 27 of the known *Serratia* viruses were scattered across 21 different clusters.

Throughout the analysis of the BZS1 genome, we identified two putative proteins presumably associated with host recognition via bacterial LPS O-antigen, namely, a tail protein (putative O-antigen deacetylase) that is probably used for attachment and an acetyltransferase, which is potentially involved in the host O-antigen conversion and could benefit the phage (facilitating detachment of progeny virions after the infection cycle), as well as the host (providing protection against superinfection). It was also shown that the BZS1-encoded DNA MTase modified GATC sequences, which mimics the specificity of Dam MTase of its host, while DNA MTase, encoded by prophage OS31, showed extremely relaxed substrate specificity and modified adenine residues, in various sequence contexts.

A unique feature discovered in the OS31 genome is the presence of an IS element, which is considered to be a fairly rare phenomenon in phages and a putative novel toxin–antitoxin system.

Finally, BZS1 is the first *Serratia* phage isolated from an oligotrophic environment and it propagates on a strain derived from the same niche. In the future, both might become a model system to provide key insights into host–bacterial interactions, where both components are from the same and rather unique environment.

## Supplementary Materials

Supplementary materials can be found at https://www.mdpi.com/1422-0067/21/18/6709/s1. Table S1. Characterization of annotated *Serratia* phage genomes available in the NCBI Viral RefSeq database. Table S2. Genes located within the vB_SspM_BZS1 phage. Table S3. Genes located within the vB_SspS_OS31 phage. Table S4. The comparison of vB_SspM_BZS1 and vB_SspS_OS31 phage-encoded proteins with *Serratia* phages using protein BLAST. Table S5. vConTACT-based neighbors of vB_SspM_BZS1 and vB_SspS_OS31 phages. Table S6. The comparison of vB_SspS_OS31 proteins with other phages. Table S7. Prophages identified within bacterial genomes similar to vB_SspM_BZS1 and vB_SspS_OS31. Table S8. List of strains used in this study. Table S9. Oligonucleotide primers used in this study. Figure S1. Comparative restriction patterns of plasmid DNAs prepared from *E. coli* cells grown in the presence or absence of inducer IPTG and cleaved with selected restriction enzymes (REases). Figure S2. Analysis of the BZS1 virion proteins by SDS-PAGE (12%). Figure S3. Electron micrographs of negatively stained OS31 virions. Figure S4. Visualization of coverage of the Illumina reads from the whole genome sequence analysis of OS31 and BZS1. Figure S5. Protein-based phage similarity network. Figure S6. The comparison of *Serratia* phage Eta with other phages. Figure S7. The all against all protein-based comparison of BZS1 and OS31 with a *Serratia marcescens* SmUNAM836 prophage.
