# Supplementary material for: Identification, Characterization, and Genomic Analysis of Novel Serratia Temperate Phages from a Gold Mine
Source: Int J Mol Sci. 2020 Sep 13;21(18):6709. doi: 10.3390/ijms21186709 (PMC7556043; doi:10.3390/ijms21186709)
Supplement: Supplementary file 1 [file ijms-21-06709-s001.zip › Nowy folder/SUPPLEMENTARY.pdf]

Supplementary materials for

Identification, Characterization and Genomic Analysis of Novel *Serratia* Temperate Phages from a Gold Mine

Katarzyna Bujak, Przemyslaw Decewicz, Jerzy Kaminski, and Monika Radlinska*

This PDF file includes:

Tables S1 to S9

Figures S1 to S7

References

Table S1. Characterization of annotated *Serratia* phage genomes available in the NCBI Viral RefSeq database.

Phage name	Size (bp)	Acc. No.	CDS	tRNA	Temperate /virulent	Family	Place of phage isolation	Host name	Place of host isolation	References
2050H1	159631	MF285619	214	0	V	*Ackermannviridae*	raw sewage	*S. marcescens* wk2050	clinical samples	[1]
2050H2	39216	MF285620	43	0	V	*Podoviridae*	no data	no data	no data	Unpublished
2050HW	276025	MF285618	363	0	V	*Myoviridae*	raw sewage	*S. marcescens* wk2050	clinical samples	[1]
3M	159398	MH929319	201	2	V	*Ackermannviridae*	polluted water	*S. marcescens* 2170c	no data	[2]
BF	357154	NC_041917	550	29	V	*Myoviridae*	composted grass	*S. marcescens* UCC2017	composted grass	[3]
CBH8	171175	MF036691	277	15	V	*Myoviridae*	sewage treatment plant	*Serratia* sp. ATCC 39006	laboratory strain (originally from channel water)	[4]
CHI14	171175	NC_041996	277	15	V	*Myoviridae*	sewage treatment plant	*Serratia* sp. ATCC 39006	laboratory strain (originally from channel water)	[4]
Eta	42724	NC_021563	69	0	T	*Siphoviridae*	Supernatant of an overnight culture of a *S. marcescens* ES	*S. marcescens* CV/rc3 (HER 1311)	Centre de Référence pour les Virus Bactériens Félix d'Hérelle	[5]
IME250	154938	NC_042047	194	4	V	*Ackermannviridae*	hospital sewage	*Serratia rubidaea*	clinical samples	[6]
JS26	63971	MN505213	84	0	V	*Siphoviridae*	no data	*Serratia* sp. ATCC 39006	laboratory strain (originally from channel water)	[7]
MAM1	157834	NC_020083	201	3	V	*Ackermannviridae*	sewage treatment plant	*S. plymuthica*, .*S. marcescens*, *Kluyvera cryocrescens*	clinical samples, enviromental samples and laboratory strains	[8]
Moabite	273933	MK994515	338	2	V	*Myoviridae*	swine farm samples	*S. marcescens* D1	clinical samples	[9]
MTx	68621	MK618717	103	0	V	*Myoviridae*	activated sludge	*S. marcescens* D1	clinical samples	[10]
Muldoon	167547	MN095771	259	4	V	*Myoviridae*	wastewater treatment plant	*S. marcescens* D1	clinical samples	[11]
MyoSmar	68745	MN062189	105	0	V	*Myoviridae*	park pond	*S. marcescens* D1	clinical samples	[12]
Parlo	62853	MK618715	87	0	T	*Podoviridae*	pooled swine, fecal and soil	*S. marcescens* D1	clinical samples	[13]
PCH45	212807	MN334766	225	0	V	*Myoviridae*	sewage	*Serratia* sp. ATCC 39006	laboratory strain (originally from channel water)	[14]
PhiZZ30	167484	MT176426	268	9	V	*Myoviridae*	no data	no data	no data	Unpublished
Pila	38678	MN098329	52	0	V	*Podoviridae*	municipal wastewater	*S. marcescens* D1	clinical samples	[15]

PS2	167266	NC_024121	276	4	V	*Myoviridae*	fishpond water	*S. marcescens* S2	clinical samples	[16]
Scaap	42969	MH553517	59	0	V	*Siphoviridae*	activated sludge	*S. marcescens*	water treatment plant	[17]
Serbin	42882	MK608336	66	0	V	*Siphoviridae*	pond water	*S. marcescens*	pond water	[18]
Slocum	112436	MN095770	168	23	V	*Siphoviridae*	municipal wastewater	*S. marcescens* D1	clinical samples	[19]
SM9-3Y	39631	KX778611	48	0	V	*Podoviridae*	raw sewage	*S. marcescens*	clinical sapmles	[20]
X20	172450	MF036692	279	17	V	*Myoviridae*	sewage treatment plant	*Serratia* sp. ATCC 39006	laboratory strain (originally from channel water)	[4]
BZS1	44995	MT843275	74	0	T	*Myoviridae*	gold and arsenic mine	*Serratia* sp. OS31	gold and arsenic mine	This work
OS31	42280	MT843276	60	0	T	*Siphoviridae*	gold and arsenic mine	*Serratia* sp. OS31	gold and arsenic mine	This work

Table S2. Genes located within the vB_SspM_BZS1 phage. Proteins identified by mass spectrometry are highlighted in bold.

ORF no.	Coding region (bp)	Strand	Protein size (aa)	Predicted function
1	75-674	+	199	terminase small subunit
2	646-2625	+	659	terminase large subunit
3	2634-2897	+	87	head-tail adapter
4	2963-4543	+	526	portal protein
5	4540-5400	+	286	head-tail preconnector protease
6	5404-6003	+	199	hypothetical protein
7	6003-6404	+	133	**head decoration protein**
8	6508-7557	+	349	**major capsid protein**
9	7559-7981	+	140	hypothetical protein
10	7981-8325	+	114	head-tail joining protein
11	8322-8864	+	180	**ATP-binding tail sheath protein**
12	8869-9063	+	64	DUF2635 domain-containing protein Mup38
13	9060-10565	+	501	**tail sheath protein, Mup39**
14	10575-10943	+	122	**tail tube protein Mup40**
15	10945-11235	+	96	tail assembly chaperone protein Mup41
16	11357-13276	+	639	tape measure protein, Mup42
17	13313-14716	+	467	**tail/DNA circulation protein, Mup43**
18	14713-15783	+	356	**baseplate wedge protein, Mup44**
19	15783-16373	+	196	**baseplate assembly protein, Mup45**
20	16370-16807	+	145	**tail protein, Mup46**
21	16811-17959	+	382	**tail protein, Mup47**
22	17956-18630	+	224	**tail protein YmfQ family**
23	18674-19162	+	162	tail protein
24	19193-20323	-	376	peptidoglycan/LPS O-acetylase
25	20333-20605	-	90	XRE family transcriptional regulator
26	20643-20816	-	57	hypothetical protein
27	20926-23307	+	793	**tail fibre/acetylesterase**
28	23308-24213	+	301	**virion-associated protein**[a]
29	24493-25656	-	387	tyrosine integrase
30	25512-25880	-	122	excisionase
31	25892-26116	-	74	hypothetical protein
32	26038-26700	-	220	DNA methyltransferase MT-A70 family
33	26703-26885	-	60	ANR family transcriptional regulator
34	26901-27281	-	126	hypothetical protein
35	27284-27493	-	69	HigB-like protein
36	27513-27731	-	71	hypothetical protein
37	27747-28016	-	89	hypothetical protein
38	28062-28763	-	233	hypothetical protein
39	28773-29027	-	84	hypothetical protein
40	29028-29564	-	178	hypothetical protein
41	29557-29748	-	63	hypothetical protein
42	29748-30230	-	160	hypothetical protein
43	30249-30518	-	89	hypothetical protein
44	30511-30774	-	87	hypothetical protein
45	30767-30988	-	73	molecular chaperone DnaJ
46	30988-31635	-	215	RNA-binding protein
47	31632-32102	-	156	hypothetical protein

48	32086-32928	-	280	ParB-like protein
49	32932-33168	-	78	hypothetical protein
50	33171-33314	-	47	hypothetical protein
51	33308-33520	-	70	hypothetical protein
52	33926-34054	-	42	hypothetical protein
53	34084-34293	-	69	hypothetical protein
54	34311-34574	-	87	hypothetical protein
55	35111-35851	-	246	repressor protein C
56	35950-36198	+	82	XRE-transcriptional regulator Cro-like
57	36216-36530	+	104	hypothetical protein
58	36539-36811	+	90	hypothetical protein
59	36826-37566	+	246	KilA-N domain-containing protein
60	37556-37738	+	60	DUF4222 domain-containing protein
61	37735-38757	+	340	DNA replication protein O
62	38754-39728	+	324	DUF968 domain-containing protein, HNH
63	39725-40081	+	118	endodeoxyribonuclease RusA
64	40078-40782	+	234	antitermination protein Q
65	41046-41453	+	135	lipoprotein RcsF-like protein
66	41621-41917	+	98	holin
67	41919-42449	+	176	lysozyme R
68	42446-42970	+	174	endopeptidase Rz
69	42543-42866	+	107	lipoprotein Rz1 precursor
70	43380-43634	+	84	DNA polymerase III theta subunit
71	43631-43771	+	46	hypothetical protein
72	43813-44457	+	214	hypothetical protein
73	44482-44727	+	81	hypothetical protein
74	44706-44930	+	74	hypothetical protein

[a] function was assigned based on proteome analysis

Table S3. Genes located within the vB_SspS_OS31 phage.

ORF no.	Coding region (bp)	Protein size (aa)	Strand	Possible function
1	263-1660	465	+	tyrosine integrase
2	1657-1872	71	-	excisionase
3	1887-2456	189	-	3'-5' exoribonuclease
4	2532-3065	177	-	hypothetical protein
5	3141-3674	177	-	hypothetical protein
6	3690-3908	72	-	HigB-like protein
7	3905-4723	272	-	ParB-like partition protein
8	4817-5626	269	-	DUF2303 family protein
9	5662-6024	120	-	hypothetical protein
10	6210-6521	103	+	hypothetical protein
11	6396-6533	45	-	hypothetical protein
12	6574-6711	45	-	hypothetical protein
13	6729-6887	52	-	DUF1391 family protein
14	7423-8181	252	-	repressor protein CI
15	8242-8436	64	+	Cro-like transcriptional regulator
16	8513-9022	169	+	repressor protein CII-like
17	9027-9326	99	+	hypothetical protein
18	9515-11041	508	+	DEAD-like helicase
19	11038-12024	328	+	DNA primase
20	12008-12586	192	+	recombination protein NinG

21	12583-13251	222	+	antiterminator Q protein
22	13288-13461	57	+	hypothetical protein
23	13602-14138	178	+	hypothetical protein
24	14138-14632	164	+	hypothetical protein
25	14864-15892	342	+	DNA adenine methyltransferase
26	15973-16314	113	+	holin
27	16301-16735	144	+	lysozyme R
28	16732-17271	179	+	endopeptidase Rz
29	16838-17161	107	+	lipoprotein Rz1 precursor
30	17268-17480	70	+	hypothetical protein
31	17501-17698	65	+	hypothetical protein
32	17805-18050	81	+	hypothetical protein
33	18110-18406	98	+	hypothetical protein
34	18403-18741	112	+	HNH endonuclease
35	18741-18941	66	+	hypothetical protein
36	19160-19624	154	+	terminase small subunit
37	19644-21314	556	+	terminase large subunit
38	21314-22582	422	+	portal protein
39	22600-23280	226	+	prohead protease
40	23282-24445	387	+	major capsid protein
41	24488-24814	108	+	head-tail connector protein
42	24814-25140	108	+	head closure protein
43	25137-25553	138	+	tail protein
44	25550-25897	115	+	DUF3160 domain-containing protein
45	25941-26873	310	-	IS3-like element IS150 family transposase B
46	26798-27310	171	-	IS3-like element IS150 family transposase A
47	27396-28118	240	+	major tail subunit
48	28136-28558	140	+	assembly chaperone
49	28555-28845	96	+	DUF 4035 domain-containing protein
50	28878-32240	1120	+	tail tape measure protein
51	32237-32590	117	+	tail minor protein
52	32599-33351	250	+	tail tip protein L
53	33354-34055	233	+	tail tip protein K
54	34099-34437	112	+	phage protein
55	34476-35090	204	+	tail assembly protein I
56	35151-38498	1115	+	tail tip protein, host specificity J
57	38538-40817	759	+	tail fiber protein
58	40810-41577	255	+	hypothetical protein
59	41600-41839	79	-	hypothetical protein
60	41839-42081	80	-	DinI family protein

Table S4. The comparison of vB_SspM_BZS1 and vB_SspS_OS31 phage-encoded proteins with *Serratia* phages using protein BLAST (1e-10 e-value threshold).

Query	Predicted function	Phage/protein Acc no.	% ident	E-value	Coverage %
PhiBZS1_p02	terminase large subunit	JS26, QGF20868.1	25,404	3,15E-18	58
JS26, QGF20868.1	terminase large subunit	PhiBZS1_p02		2,64E-18	75
PhiBZS1_p04	phage portal protein	JS26, QGF20866.1	30,577	5,63E-73	95
JS26, QGF20866.1	hypothetical protein	PhiBZS1_p04		6,07E-73	91
PhiBZS1_p05	head-tail preconnector protease	JS26, QGF20871.1	33,099	6,40E-48	95
JS26, QGF20871.1	hypothetical protein	PhiBZS1_p05		6,70E-34	64
PhiBZS1_p08	major capsid protein	JS26, QGF20877.1	28,797	4,42E-30	86
JS26, QGF20877.1	hypothetical protein	PhiBZS1_p08		4,48E-30	84
PhiBZS1_p35	HigB-like protein	PhiOS31_p06	37,097	7,53E-12	90
PhiOS31_p06	HigB-like protein	PhiBZS1_p35			86
PhiBZS1_p038	hypothetical protein	Parlo, QBQ72186.1	88,976	1,91E-83	55
Parlo, QBQ72186.1	hypothetical protein	PhiBZS1_p38		2,00E-83	52
PhiBZS1_p45	molecular chaperone DnaJ	Parlo, QBQ72187.1	75	1,83E-29	77
Parlo, QBQ72187.1	molecular chaperone	PhiBZS1_p45		1,78E-29	79
PhiBZS1_p46	RNA-binding protein	Eta, YP_008130315.1	47,257	1,38E-53	99
Eta, YP_008130315.1	hypothetical protein	PhiBZS1_p46		1,46E-53	97
PhiBZS1_p48	ParB-like partitioning protein	Eta,YP_008130295.1	55,474	7,9E-102	95
Eta,YP_008130295.1	putative parB-like partition protein	PhiBZS1_p48		1,18E-89	99
PhiBZS1_p48	ParB-like partitioning protein	PhiOS31_p07	35,484	5,01E-44	89
PhiOS31_p07	ParB-like partitioning protein	PhiBZS1_p48		2,82E-37	90
PhiBZS1_p49	hypothetical protein	Parlo, QBQ72162.1	69,014	1,24E-32	88
Parlo, QBQ72162.1	hypothetical protein	PhiBZS1_p49		1,16E-32	97
PhiBZS1_p68	endopeptidase Rz	PhiOS31_p28	77,108	1,15E-93	95
PhiOS31_p28	endopeptidase Rz	PhiBZS1_p68		1,18E-93	93
PhiOS31_p07	ParB-like partitioning protein	Eta, YP_008130295.1	31,474	1,11E-26	88
Eta, YP_008130295.1	putative parB-like partition protein	PhiOS31_p07			90
PhiOS31_p17	hypothetical protein	Eta, YP_008130291.1	41,791	9,88E-15	68
Eta, YP_008130291.1	hypothetical protein	PhiOS31_p17		8,38E-15	80
PhiOS31_p18	DEAD-like helicase	Eta, YP_008130293.1	23,81	1,18E-17	77
Eta, YP_008130293.1	putative helicase	PhiOS31_p18	23,853	1,52E-17	62
PhiOS31_p18	DEAD-like helicase	PS2, YP_009030245.1	23,1	3,60E-12	61
PS2, YP_009030245.1	putative split helicase	PhiOS31_p18		3,53E-12	64
PhiOS31_p27	lysozyme R	Eta, YP_008130340.1	51,007	5,07E-46	100
Eta, YP_008130340.1	lysin	PhiOS31_p27		5,28E-46	99
PhiOS31_p51	tail minor protein	Scapp, AXH50962.1	40,4	1,20E-57	96
Scapp, AXH50962.1	putative minor tail protein	PhiOS31_p51		1,23E-57	96
PhiOS31_p52	tail tip protein L	Scapp, AXH50963.1	40	1,12E-50	98
Scapp, AXH50963.1	putative minor tail protein	PhiOS31_p52		1,19E-50	92
PhiOS31_p54	phage protein	Scapp, AXH50964.1	43,455	7,61E-52	94
Scapp, AXH50964.1	putative minor tail protein	PhiOS31_p54	44,792	5,91E-49	97
PhiOS31_p55	tail assembly protein I	Scapp, AXH50965.1	38,046	0	86
Scapp, AXH50965.1	putative tail tip fiber protein	PhiOS31_p55		0	87
PhiOS31_p57	tail fibre/acetyltransferase	Serbin, QBQ72942.1	33,291	5,5E-107	99
Serbin, QBQ72942.1	putative tail fiber protein	PhiOS31_p57	32,997	1,4E-100	99
PhiOS31_p57	tail fibre/acetyltransferase	PhiBZS1_p27	32,824	4,46E-11	16

Table S5. vConTACT-based neighbors of vB_SspM_BZS1 and vB_SspS_OS31 phages. The table shows the results of proteome-based phages clustering and the resulting assignment of viral clusters and status.

**vB_SspM_BZS1 neighbor nodes**						
**Family**	**vConTACT viral cluster**	**vConTACT status**	**Phage name**	**Host name**	**Genome accession number**	**Reference**
Myoviridae	194_0	Clustered	*Enterobacteria* phage phiP27	*Escherichia coli* ONT:H- strain 2771/97	AJ298298.1	[21]
Myoviridae	-	Outlier	*Enterobacteria* phage SfI	*Shigella* flexneri serotype 1a strain 019	JX509734.1	[22]
Myoviridae	194_0	Clustered	*Enterobacteria* phage SfV	*Shigella flexneri*	U82619.2	[23]
Myoviridae	194_0	Clustered	*Yersinia* phage YeP2	*Yersinia* enteroco*li*tica BJ2014-1008297168	MK733260.1	unpublished
Myoviridae	194_0	Clustered	*Yersinia* phage YeP3	*Yersinia* enteroco*li*tica QH2012-y91	MK733261.1	unpublished
Myoviridae	194_0	Clustered	*Yersinia* phage YeP1	*Yersinia* enteroco*li*tica BJ2014-1008329276	MK733259.1	unpublished
Myoviridae	-	Overlap (VC_379/VC_385)	*Escherichia* phage 1720a-02	*Escherichia coli* strain O174:H21 isolate 1720a	KF030445.1	unpublished
Myoviridae	-	Outlier	Pseudomonas phage DVM-2008	*Pseudomonas fluorescens* Q8r1-96	EU982300.1	[24]
Myoviridae	30_0	Clustered	*Klebsiella* phage ST15-VIM1phi2.1	*Klebsiella pneumoniae* ST15-VIM1	MK448228.1	unpublished
Myoviridae	-	Outlier	Phage NV21	unkown; viral metagenome	MK047641.1	unpublished
Myoviridae	-	Outlier	*Pectobacterium* phage ZF40	*Pectobacterium carotovorum*	JQ177065.1	[25]
Myoviridae	194_0	Clustered	*Salmonella* phage 118970_sal3	*Salmonella* enterica	KU927493.2	[26]
Myoviridae	194_0	Clustered	*Salmonella* phage ST64B	*Salmonella* enterica subsp. enterica ser. Typhimurium	AY055382.1	[27]
Myoviridae	194_0	Clustered	*Shigella* phage SfII	*Shigella* flexneri Serotype 2a	KC736978.1	[28]
Myoviridae	194_0	Clustered	*Shigella* phage SfIV	*Shigella* flexneri serotype 4av	KC814930.1	[29]
Siphoviridae	75_1	Clustered	*Klebsiella* phage ST13-OXA48phi12.5	*Klebsiella pneumoniae* ST13-OXA48	MK714353.1	unpublished
Myoviridae	-	Outlier	vB_SspM_BZS1	*Serratia* sp. OS31	MT843275	This work
**vB_SspS_OS31 neighbor nodes**						
**Family**	**vConTACT viral cluster**	**vConTACT status**	**Phage name**	**Host name**	**Genome accession number**	**References**
Siphoviridae	-	Overlap (VC_194/VC_247)	*Escherichia* virus HK022	*Escherichia coli* K12	AF069308.1	[30]
Siphoviridae	245_0	Clustered	*Burkholderia* virus phiE125	*Burkholderia thailandensis* E125	AF447491.1	[31]
Siphoviridae	282_1	Clustered	*Enterobacteria* phage BP-4795	*Escherichia coli*, strain 4795/97, serotype O84:H4	AJ556162.1	[32]
Siphoviridae	245_0	Clustered	*Burkholderia* virus phi1026b	*Burkholderia* pseudomallei 1026b	AY453853.1	[33]
Siphoviridae	-	Overlap (VC_279/VC_378)	*Klebsiella* phage phiKO2	*Klebsiella oxytoca*	AY374448.1	[34]

Siphoviridae	-	Overlap (VC_194/VC_247/VC_283)	*Escherichia* virus HK97	*Escherichia coli* K12	AF069529.1	[30]
Siphoviridae	-	Outlier	*Burkholderia* phage Bcep176	*Burkholderia cepacia*	DQ203855.1	unpublished
Siphoviridae	282_1	Clustered	Stx2-converting phage 1717	*Escherichia coli* O157:H7	FJ188381.1	[35]
Siphoviridae	282_1	Clustered	*Enterobacteria* phage YYZ-2008	*Escherichia coli* EC970520 O157:H7	FJ184280.1	unpublished
Siphoviridae	-	Outlier	*Burkholderia* phage KS9	*Burkholderia pyrrocinia* strain LMG 21824	FJ982340.1	[36]
Siphoviridae	245_0	Clustered	*Burkholderia* virus phi6442	*Burkholderia pseudomallei*	CP000625.2	unpublished
Siphoviridae	-	Outlier	*Escherichia* phage HK639	*Escherichia coli*	HM208537.1	unpublished
Siphoviridae	-	Overlap (VC_194/VC_247/VC_283)	*Escherichia* phage HK75	*Escherichia coli* K-12	HM173637.1	unpublished
Siphoviridae	-	Outlier	*Cronobacter* phage phiES15	*Cronobacte sakazakii* ES15	JQ780327.1	[37]
Siphoviridae	-	Overlap (VC_194/VC_247/VC_283)	*Enterobacteria* phage HK140	*Escherichia coli*	JQ086370.1	unpublished
Siphoviridae	-	Overlap (VC_194/VC_247/VC_283)	*Enterobacteria* phage HK633	*Escherichia coli*	JQ086377.1	unpublished
Siphoviridae	-	Overlap (VC_377/VC_379/VC_385)	*Enterobacteria* phage mEp237	*Escherichia coli*	JQ182730.1	unpublished
Siphoviridae	-	Overlap (VC_194/VC_247/VC_283)	*Enterobacteria* phage HK542	*Escherichia coli*	JQ086373.1	unpublished
Siphoviridae	-	Overlap (VC_194/VC_247/VC_283)	*Enterobacteria* phage HK446	*Escherichia coli*	JQ086372.1	unpublished
Siphoviridae	-	Overlap (VC_194/VC_247/VC_283)	*Enterobacteria* phage mEpX1	*Escherichia coli*	JQ182727.1	unpublished
Siphoviridae	-	Overlap (VC_194/VC_247/VC_283)	*Enterobacteria*l phage mEp234	*Escherichia coli*	JQ182732.1	unpublished
Siphoviridae	-	Overlap (VC_377/VC_379/VC_385)	*Enterobacteria* phage HK225	*Escherichia coli*	JQ086371.1	unpublished
Siphoviridae	-	Overlap (VC_194/VC_247/VC_283)	*Enterobacteria* phage HK544	*Escherichia coli*	JQ086374.1	unpublished
Siphoviridae	-	Overlap (VC_194/VC_247/VC_283)	*Enterobacteria* phage mEp235	*Escherichia coli*	JQ182731.1	unpublished
Siphoviridae	-	Overlap (VC_194/VC_247/VC_283)	*Enterobacteria* phage mEpX2	*Escherichia coli*	JQ182726.1	unpublished
Siphoviridae	196_0	Clustered	*Enterobacteria*l phage mEp390	*Escherichia coli*	JQ182729.1	unpublished
Siphoviridae	-	Overlap (VC_194/VC_247/VC_283)	*Enterobacteria* phage HK106	*Escherichia coli*	JQ086369.1	unpublished
Siphoviridae	196_0	Clustered	*Cronobacter* phage ENT39118	*Cronobacter sakazakii*	HQ201307.1	[38]
Siphoviridae	282_1	Clustered	*Enterobacteria* phage 2851	*Escherichia coli* O157:H7 strain CB2851	FM180578.1	[39]

Siphoviridae	282_1	Clustered	Stx2-converting phage Stx2a_F349	*Escherichia coli* O157:H7 strain F349	AP012530.1	[40]
Siphoviridae	282_0	Clustered	Stx2-converting phage Stx2a_1447	*Escherichia coli* O157:H7 strain 1447	AP012536.1	[40]
Siphoviridae	282_0	Clustered	Stx2-converting phage Stx2a_WGPS2	*Escherichia coli* O157:H7 strain 980938	AP012537.1	[40]
Siphoviridae	282_1	Clustered	Stx2-converting phage Stx2a_WGPS4	*Escherichia coli* O157:H7 strain 990281	AP012538.1	[40]
Siphoviridae	282_1	Clustered	Stx2-converting phage Stx2a_WGPS6	*Escherichia coli* O157:H7 strain 990570	AP012539.1	[40]
Siphoviridae	282_1	Clustered	Stx2-converting phage Stx2a_WGPS8	*Escherichia coli* O157:H7 strain 982243	AP012540.1	[40]
Siphoviridae	196_0	Clustered	*Klebsiella* phage 1 LV-2017	*Klebsiella pneumoniae* KH43	KY271401.1	[41]
Siphoviridae	196_0	Clustered	*Morganella* phage IME1369_02	*Morganella morganii* IME1369	KY653119.1	unpublished
Siphoviridae	-	Overlap (VC_194/VC_247/VC_283)	*Escherichia* phage ECP1	*Escherichia coli* MG1655	KY979108.1	unpublished
Siphoviridae	282_1	Clustered	*Escherichia* phage JLK-2012	*Escherichia coli* O55:H7 str. DEC5E	JQ347801.1	[42]
Siphoviridae	196_0	Clustered	*Escherichia* phage phi467	*Escherichia coli* strain EF467	LN997803.1	unpublished
Siphoviridae	-	Outlier	*Salmonella* phage FSL SP-016	*Salmonella enterica*	KC139516.1	[43]
Siphoviridae	196_0	Clustered	*Aeromonas* phage AsXd-1	*Aeromonas salmonicida*	MH178096.1	[44]
Siphoviridae	-	Outlier	*Escherichia* phage PHB10	*Escherichia coli*	MK524177.1	[45]
Siphoviridae	196_0	Clustered	*Klebsiella* phage ST13-OXA48phi12.2	*Klebsiella pneumoniae* ST13-OXA48	MK422452.1	[46]
Siphoviridae	196_0	Clustered	*Klebsiella* phage ST899-OXA48phi17.2	*Klebsiella pneumoniae* ST899-OXA48	MK433583.1	[46]
Siphoviridae	196_0	Clustered	*Klebsiella* phage ST147-VIM1phi7.2	*Klebsiella pneumoniae* ST147-VIM1	MK448232.1	[46]
Siphoviridae	-	Overlap (VC_43/VC_197)	*Klebsiella* phage ST13-OXA48phi12.4	*Klebsiella pneumoniae* ST13-OXA48	MK422450.1	[46]
Siphoviridae	196_0	Clustered	*Klebsiella* phage ST101-KPC2phi6.3	*Klebsiella pneumoniae* ST101-KPC2	MK416017.1	[46]
Siphoviridae	-	Outlier	*Bordetella* phage vB_BbrS_PHB09	*Bordetella bronchiseptica* Bb01	MN103401.1	[47]
Siphoviridae	196_0	Clustered	*Enterobacter* phage LAU1	*Enterobacter hormaechei* subsp. xiangfangensis	MN688132.1	unpublished
Siphoviridae	196_0	Clustered	vB_SspS_OS31	*Serratia* sp. OS31	MT843276	This work

Table S6. The comparison of vB_SspS_OS31 proteins with other phages. The comparison was based on BLASTP searches with e-value ≤1e-15 and query coverage of HSP ≥75%. Results above the thresholds were marked as significantly similar and the cell in the table was marked with a number (1), otherwise the cell was left empty.

**vB_SspS_OS31**	**HQ201307.1**	**JQ182729.1**	**KY271401.1**	**KY653119.1**	**LN997803.1**	**MH178096.1**	**MK416017.1**	**MK422452.1**	**MK433583.1**	**MK448232.1**	**MN688132.1**	**Total amount**
**PhiOS31_p03 3'-5' exoribonuclease**				1				1				**2**
**PhiOS31_p06 HigB-like protein**						1						**1**
**PhiOS31_p07 ParB-like partition protein**		1						1				**2**
**PhiOS31_p08 DUF2303 family protein**								1				**1**
**PhiOS31_p09 hypothetical protein**								1				**1**
**PhiOS31_p14 repressor protein CI**	1	1		1			1	1				**5**
**PhiOS31_p17 hypothetical protein**					1							**1**
**PhiOS31_p18 DEAD-like helicase**	1				1			1				**3**
**PhiOS31_p19 DNA primase**	1				1			1				**3**
**PhiOS31_p25 DNA adenine methyltransferase**				1								**1**
**PhiOS31_p27 lysozyme R**				1	1							**2**
**PhiOS31_p34 HNH endonuclease**	1	1				1			1	1		**5**
**PhiOS31_p36 terminase small subunit**			1	1	1			1	1	1		**6**
**PhiOS31_p37 terminase large subunit**	1	1	1	1	1	1	1	1	1	1	1	**11**
**PhiOS31_p38 portal protein**	1	1	1	1	1	1	1	1	1	1	1	**11**
**PhiOS31_p39 prohead protease**	1	1			1	1	1				1	**6**
**PhiOS31_p40 major capsid protein**	1	1				1			1	1	1	**6**
**PhiOS31_p41 head-tail connector protein**	1	1				1					1	**4**
**PhiOS31_p42 head closure protein**	1	1	1	1	1	1	1	1	1	1	1	**11**
**PhiOS31_p43 tail protein**	1	1	1	1		1	1	1	1	1	1	**10**
**PhiOS31_p44 DUF3160 domain-containing protein**	1	1		1	1	1	1	1	1	1	1	**10**
**PhiOS31_p48 assembly chaperone**	1	1	1	1	1	1	1	1	1	1	1	**11**
**PhiOS31_p49 DUF 4035 domain-containing protein**		1	1	1	1	1	1	1	1	1	1	**10**
**PhiOS31_p50 tail tape measure protein**	1	1			1							**3**
**PhiOS31_p51 tail minor protein**		1		1		1					1	**4**
**PhiOS31_p52 tail tip protein L**	1	1		1	1	1					1	**6**
**PhiOS31_p53 tail tip protein K**	1	1		1		1					1	**5**
**PhiOS31_p55 tail assembly protein I**	1	1		1		1					1	**5**
**PhiOS31_p56 tail tip protein, host specificity J**		1		1		1					1	**4**
**PhiOS31_p60 DinI family protein**		1				1						**2**
**Total amount**	**17**	**20**	**7**	**17**	**14**	**18**	**9**	**15**	**10**	**10**	**15**	**152**

Table S7. Prophages identified within bacterial genomes similar to vB_SspM_BZS1 and vB_SspS_OS31 (minimum query coverage 24% threshold). When the nucleotide sequence of a prophage predicted by us, overlapped the prophage regions determined by authors of publications appropriate coordinates were introduced in the References column.

Host	**Genome acc. no (coordinates)**	**Family**	**Size (bp)**	**GC %**	CDS	**Integration site**	**Query Coverage, identities**	**References**
**Prophages similar to BZS1**								
Pectobacterium parmentieri IFB5605	CP026984.1 (1406812-1449822)	*Myoviridae*	43011	47.8	56	tRNA-Arg(tct) ATGGTACGCCCTACAGGGCTCGAACCTGTGACCTACGGCTTAGAAG	Query Coverage: 37% Identities: 75%	[48], 1393802 - 1457622
Serratia marcescens 4201	CP047679.1 (1216827-1261080)	*Myoviridae*	44254	52.1	60	TTCCAGTTACGAA	Query Coverage: 48% Identities: 81%	Unpublished
Serratia marcescens EL1	CP027796.1 (5072061-5116746)	*Myoviridae*	44868	52.0	59	tRNA-Leu(taa) ATTTAAAATCCCTCGGCTTATGGCTGTGCGGGTTCAAGTCCCGCCCCGGGTACCAGGGAACAAAAATACCGAATAATCAAAGCAATAAGTAGTAATGTCGTAGACCGCCGAGAGGCGGTTTTTTTGTTT	Query Coverage: 51% Identities: 82,4%	Unpublished
Serratia marcescens KS10	CP027798.1 (2825136-2869821)	*Myoviridae*	45686	52.0	59	tRNA-Leu(taa) ATTTAAAATCCCTCGGCTTATGGCTGTGCGGGTTCAAGTCCCGCCCCGGGTACCAGGGAACAAAAATACCGAATAATCAAAGCAATAAGTAGTAATGTCGTAGACCGCCGAGAGGCGGTTTTTTTGTTT	Query Coverage: 51% Identities: 82,41%	Unpublished
Serratia marcescens SmUNAM836	CP012685.1 (3321604-3366650)	*Myoviridae*	45047	52.3	56	ferredoxin-like protein YfhL GCTCGCACATGTCGCAGTTGAT	Query Coverage: 52% Identities: 82,44%	[49], 3321802-3366386
Serratia marcescens WVU-001	CP041122.1 (1032870-1079228)	*Myoviridae*	46359	52.0	64	CATCAGACTGTTTCTCTGATGCT	Query Coverage: 50% Identities: 81,84%	Unpublished
Serratia marcescens WVU-003	CP041124.1 (1032870-1079228)	*Myoviridae*	46359	52.0	64	CATCAGACTGTTTCTCTGATGCT	Query Coverage: 50% Identities: 81,84%	Unpublished
Serratia sp. 1D1416 pp1	CP032738.1 (4585981-4632239)	*Myoviridae*	46259	52.7	69	AAAAAAAGCCCCGGCAAAACGCCGG	Query Coverage: 49% Identities: 86,3%	[50]
Escherichia coli CV839-15	CP024978.1 (4348878 – 4394136)	*Myoviridae*	45259	52.1	65	DUF72 domain-containing protein CACGCAGTTAAAGTGGCGGGC	Query Coverage: 34% Identities: 70,92%	[51]
**Prophages similar to OS31**								
Serratia liquefaciens ATCC 27592	CP006252.1 (3958703-4001776)	*Siphoviridae*	43074	49.3	52	ferredoxin-like protein YfhL TCGCACATGTCGCAGTTGATGCA	Query Coverage: 47% Identities: 93,29%	[52]
Serratia liquefaciens FG3	CP033893.1 (4127044-4168355)	*Siphoviridae*	41312	52.4	55	protein EttA GGTATAGACGTATTGAGCCACGGTGCTCTA	Query Coverage: 44% Identities: 92,28%	[53]
Serratia liquefaciens JL02	CP048043.1 (4016455-4068130)	*Siphoviridae*	51676	51.0	58	TTGCGGGCTTTTTTGTGTCTGTAG	Query Coverage: 47% Identities: 93,37%	Unpublished
Serratia liquefaciens S1	CP048784.1 (4015813-4058542)	*Siphoviridae*	42730	49.9	50	ferredoxin-like protein YfhL TCGCACATGTCGCAGTTGAT	Query Coverage: 45% Identities: 93%	[54]
Serratia marcescens AR_0091	CP027533.1 (3057431-3099123)	*Siphoviridae*	41693	52.2	53	GTAATCGTGAATGTA	Query Coverage: 41% Identities: 83,7%	Unpublished

Serratia marcescens AR_0130	CP028947.1 (3408429-3448305)	*Myoviridae*	39877	51.0	54	GTAATCGTGAATGTA	Query Coverage: 24% Identities: 83,78%	Unpublished
Serratia marcescens AS1	CP010584.1 (1760327-1802678)	*Siphoviridae*	42352	52.4	49	ferredoxin-like protein YfhL CGCACATGTCGCAGTTGAT	Query Coverage: 36% Identities: 84,7%	[55]
Serratia marcescens B3R3	CP013046.2 (3636385-3679019)	*Siphoviridae*	42640	52.3	58	GTAATCGTGAATGTA	Query Coverage: 37% Identities: 84,63%	Unpublished
Serratia marcescens FDAARGOS_65	CP026050.1 (4195148-4236021)	*Myoviridae*	40874	52.8	50	ferredoxin-like protein YfhL TCGCACATATCGCAGTTGATGCA	Query Coverage: 29% Identities: 83,59%	Unpublished
Serratia marcescens SmUNAM836	CP012685.1 (3321604-3366650)	*Myoviridae*	45047	52.3	56	ferredoxin-like protein YfhL GCTCGCACATGTCGCAGTTGAT	Query Coverage: 29% Identities: 84,92%	3321802-3366386
Serratia plymuthica 4Rx13	CP006250.1 (4060625-4104885)	*Siphoviridae*	44281	49.4	44	ferredoxin-like protein YfhL GCTCGCACATATCGCAGTTGATGCA	Query Coverage: 60% Identities: 88,41%	[56], 4060600 – 4104861
Serratia quinivorans PKL12	CP038467.1 (4692222-4730406)	*Siphoviridae*	38185	52.1	53	tRNA-Gly(tcc) TGGTGGAGCTGGGGGGATTTGAACCC	Query Coverage: 53% Identities: 95,33%	Unpublished
Serratia sp. 1D1416 pp2	CP032738.1 (2691003-2735611)	*Siphoviridae*	44609	51.6	55	tRNA-Arg(tct) TGGTACGCCCTACAGGGCTCGAACCTG TGACCTACGGCTTAGAAGG	Query Coverage: 38% Identities: 86,13%	[50]
Serratia sp. JKS000199	LT907843.1 (3196614-3235487)	*Siphoviridae*	38874	52.6	46	ferredoxin-like protein YfhL CGCACATGTCGCAGTTGAT	Query Coverage: 36% Identities: 84,24%	Unpublished

Table S8. List of strains used in this study. All the strains are included in the collection of microorganisms of the Institute of Microbiology at the University of Warsaw (Poland).

Strain	Source of isolation	Use in this study	Source/ References
Escherichia coli TOP10F′	laboratory strain	Host strain for gene cloning	Invitrogen, Waltham, MA, USA
E. coli ER2566	laboratory strain	Host strain for gene expression	New England BioLabs, Ipswich, MA
E. coli ER2929 Dam⁻	laboratory strain	Host strain for gene expression	[57]
Serratia sp. OS31	gold and arsenic mine	Host strain for BZS1	Obtained from Ł. Drewniak
Serratia sp. BZSmr3	gold and arsenic mine	as a potential host for phages	This work
Serratia sp. BZSmr6	gold and arsenic mine	as a potential host for phages	This work
Yesinia enterolitica 2/O:9	clinical strain	as a potential host for phages	[58]
Escherichia coli strain 211_ECOL	clinical strain	as a potential host for phages	[59]
Serratia macescens strain 666_SMAR	clinical strain	as a potential host for phages	[59]
Serratia macescens MK	food products	as a potential host for phages	Obtained from D. Korsak
Salmonella enterica ser. Typhimurium 2119	food products	as a potential host for phages	Obtained from D. Korsak
Salmonella infantis 2182	food products	as a potential host for phages	Obtained from D. Korsak
Salmonella enterica ser. Virchow 2157	food products	as a potential host for phages	Obtained from D. Korsak
Salmonella enterica ser. Saintpaul 2015	food products	as a potential host for phages	Obtained from D. Korsak
Salmonella enterica ser. Enteritidis 1740	food products	as a potential host for phages	Obtained from D. Korsak
Salmonella sp. type Oranienburg 2016	food products	as a potential host for phages	Obtained from D. Korsak
Cronobacter sakazakii MK_1	food products	as a potential host for phages	Obtained from D. Korsak
Cronobacter turicensis MK_3	food products	as a potential host for phages	Obtained from D. Korsak
Cronobacter malonaticus MK_11	food products	as a potential host for phages	Obtained from D. Korsak
Cronobacter dublinensis MK_8	food products	as a potential host for phages	Obtained from D. Korsak
Cronobacter muytjensii MK_5	food products	as a potential host for phages	Obtained from D. Korsak

Table S9. Oligonucleotide primers used in this study.

Name of the primer	Primer seqence (5′→3′)[1]	Restriction site created and used for cloning	Plasmid vector and restriction sites used for cloning	Name of the construct
BZS1attLf	CCTGTCATGAGTGAAATAGCGAGCTCTG	-	pUC/SmaI	pKS_BZS1attL
BZS1attLr	GTTGGGAATCGATAGAAATGGTGCGTAG	-		
BZS1attRf	CTATATCTGCATACTGCTGATGAGTCG	-	pUC/SmaI	pKS_BZS1attR
BZS1attRr	GATACGATCGAGAAAGCGACAATGC	-		
cosBZS1f	CACCGTGCTCGTATACGTAC	-	pUC/SmaI	pKS_cosBZS1
cosBZS1r	GTTACCTGACTTGTTACCCGAG	-		
MetBZS1f	GTTGTT**CATATG**GCCTATCAACTTATCTATGC	NdeI	pET30a NdeI/XhoI	pET_PhiBZS1_p32
MetBZS1r	GTTGTT**CTCGAG**GTAGTTCTCTTGCTGTGCTAC	XhoI		
MetOS31f	GTTGTT**CATATG**TCAATGGTCACAAGCGAAC	NdeI	pET30a NdeI/XhoI	pET_PhiOS31_p25
MetOS31r	GTTGTT**CTCGAG**GTTACTCGGGAAGATTTCCCG	XhoI		
MinCF	CTCGTTGGTAAGATGCTGGATAAG	-	-	-
MinCr	GCTTATTGTGAAGAGCCATAATACC	-	-	-
MCf	GTCATGAGGGTTATGGTCTTAACC	-	-	-
MCr	GCAAGGCCATCGTAATGCGG	-	-	-

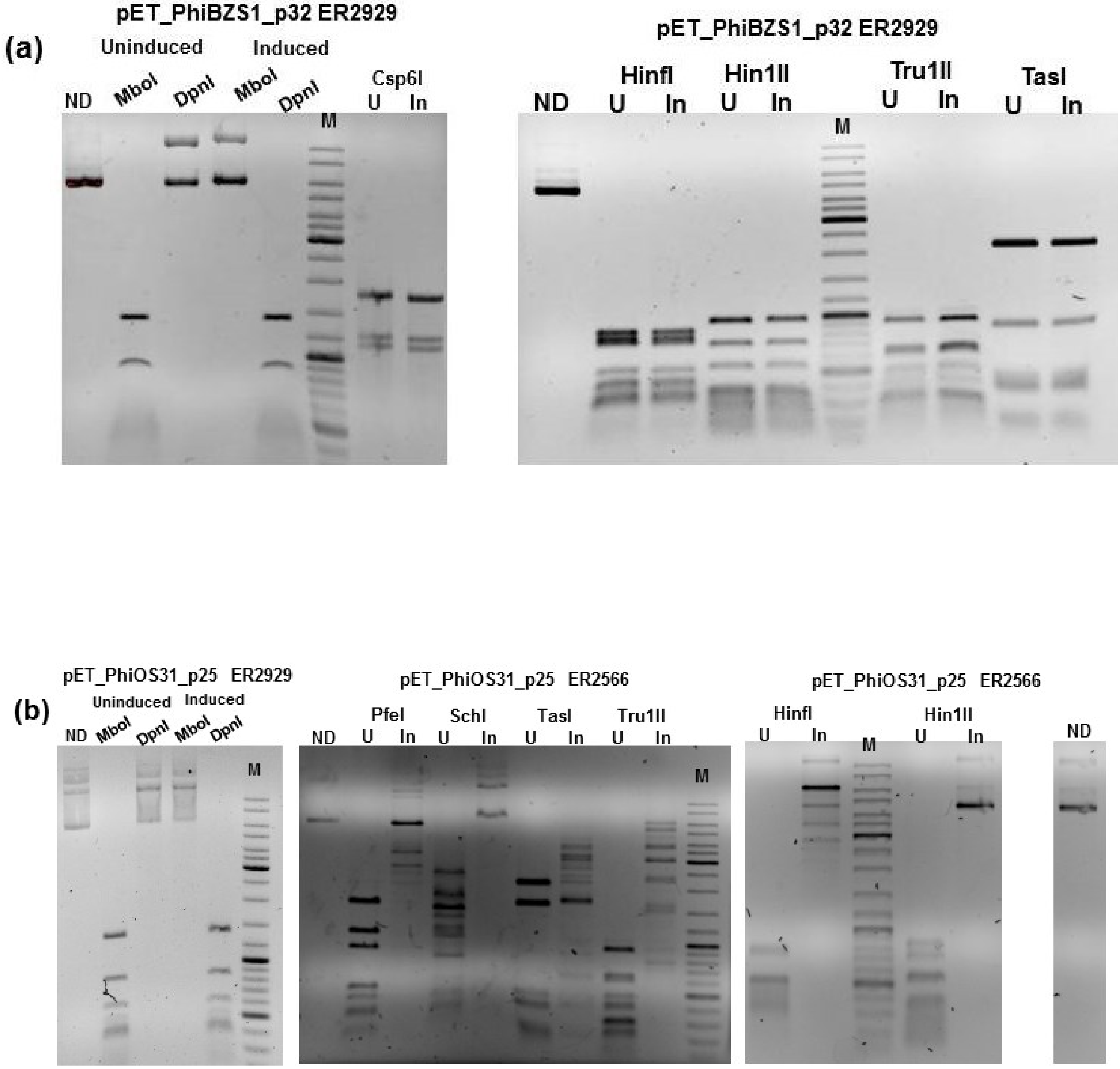

Figure S1. Comparative restriction patterns of plasmid DNAs prepared from *E. coli* cells grown in the presence or absence of inducer IPTG and cleaved with selected restriction enzymes (REases). Comparison of the restriction patterns of the plasmid pET_PhiBZS1_p32 DNA isolated from the *E. coli* ER2929 cells grown in the presence (In) or absence (U) of the inducer (IPTG) and cleaved with the selected REases **(a)**; the plasmid pET_PhiOS31_p25 isolated from the *E. coli* ER2929 cells grown in the presence (In) or absence (U) of the inducer (IPTG) and cleaved with the selected REases and the plasmid pET_PhiOS31_p25 isolated from the *E. coli* ER2566 cells grown in the presence (In) or absence (U) of the inducer (IPTG) and cleaved with the selected REases **(b)**. Digest mixtures were electrophoresed on 0.9% agarose gels and stained with ethidium bromide. ND-undigested, DNA, M – GeneRuler DNA Ladder Mix 100-10,000 bp size marker (Thermo Fisher Scientific).

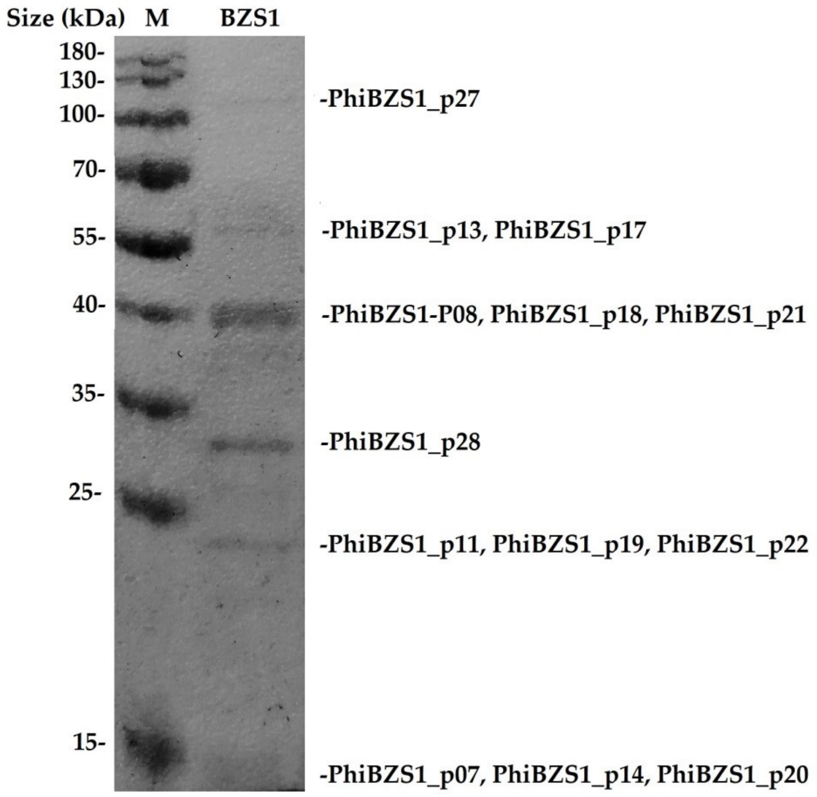

Figure S2. Analysis of the BZS1 virion proteins by SDS-PAGE (12%). Lane M, Page Ruler protein ladder SM0671 (Thermo Scientific). Proteins identified by mass spectrometry are shown on the right.

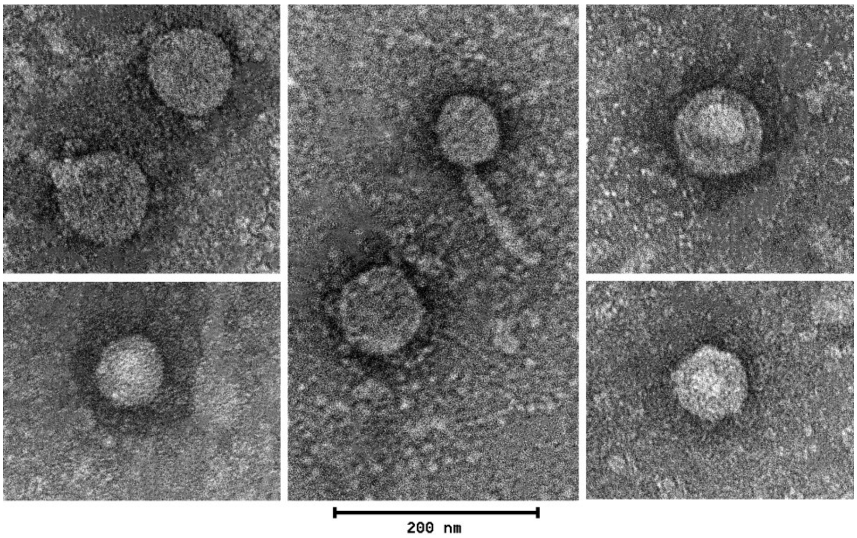

Figure S3. Electron micrographs of negatively stained OS31 virions. The scale bar in the transmission electron microscopy image represents 200 nm.

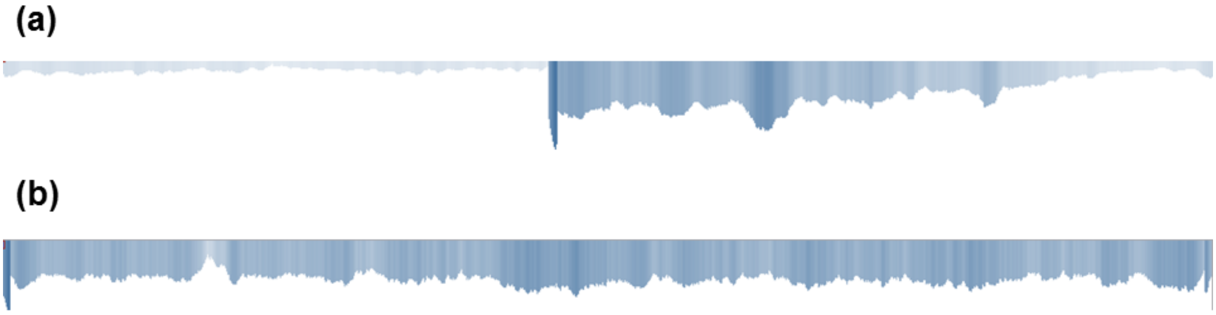

Figure S4. Visualization of coverage of the Illumina reads from the whole genome sequence analysis of OS31 **(a)** and BZS1 (as a control) **(b)** using Tablet [60,61] as graphical viewer. The Sequence Alignment/Map (SAM) formats were prepared using web-based platform Galaxy (https://usegalaxy.org/). Presented coverage ranges to c.a. 1600x and 500x in case of OS31 and BZS1, respectively.

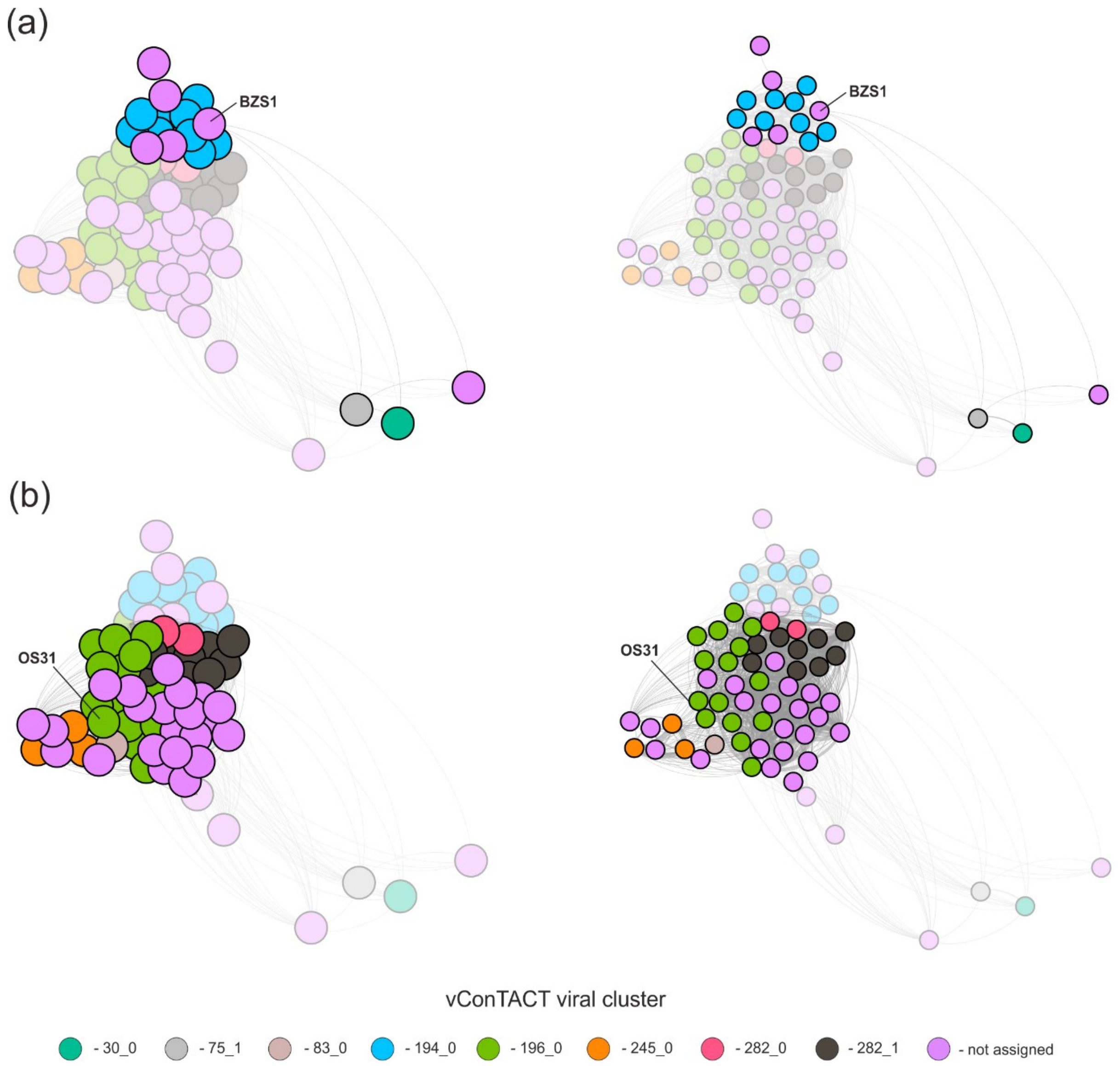

Figure S5. Protein-based phage similarity network. The networks representing BZS1 **(a)** and OS31 **(b)** phages with their neighbors were presented in a dense (left panels; as shown on Figure 4) and relaxed (right panels) layout. The nodes were colored based on the vConTACT v0.93 viral clusters.

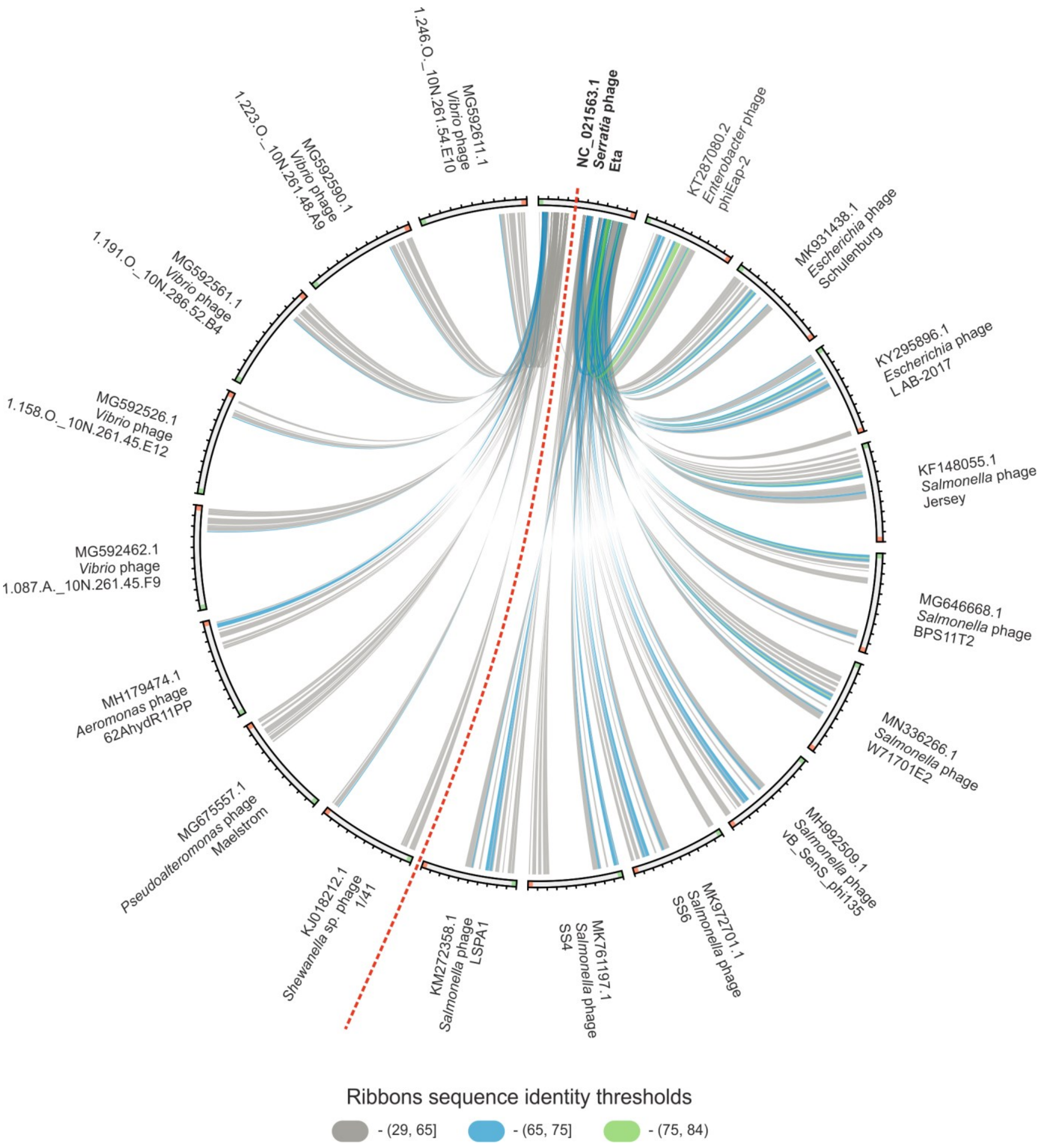

Figure S6. The comparison of *Serratia* phage Eta with other phages. The comparison was based on the protein sequence identity shown with ribbons according the legend. The anchors of ribbons correspond to protein-encoding genes location. Green and orange rectangles at the ends of ideograms reflect the beginning and end of phage genome.

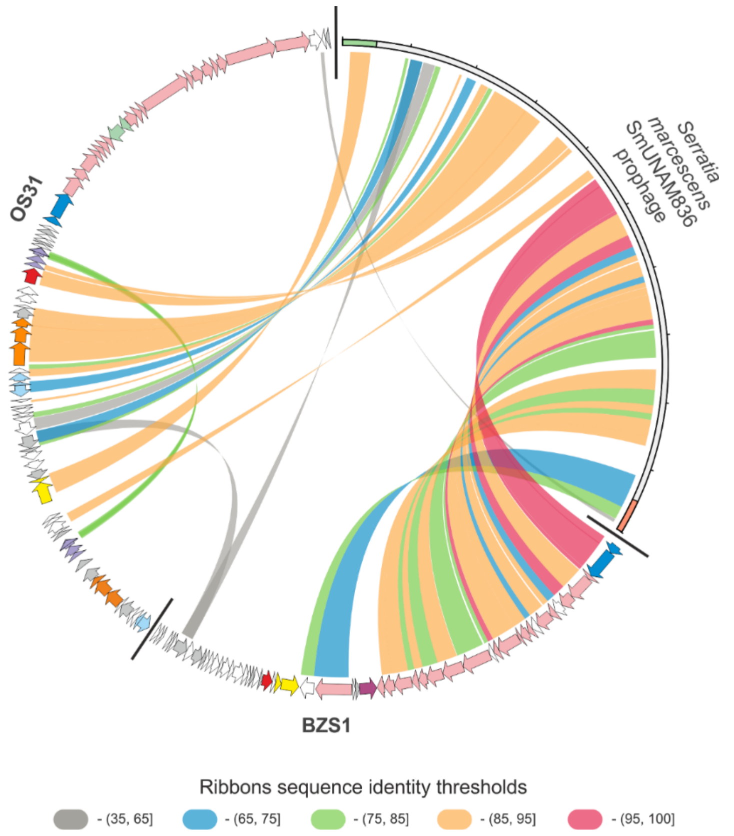

Figure S7. The all against all protein-based comparison of BZS1 and OS31 with a *Serratia marcescens* SmUNAM836 prophage (CP012685.1, coordinates: 3321604-3366650)**.** Ribbons indicate sequence identity as shown on the legend. The anchors of ribbons correspond to protein-encoding genes location. Green and orange rectangles at the ends of *S. marcescens* SmUNAM836 prophage ideogram reflect the beginning and end of the prophage genome.

References

1. Tian, C., et al., *Identification and molecular characterization of Serratia marcescens phages vB_SmaA_2050H1 and vB_SmaM_2050HW.* Arch Virol, 2019. **164**(4): p. 1085-1094.
2. Regué, M., C. Fabregat, and M. Viñas, *A generalized transducing bacteriophage for Serratia marcescens.* Res Microbiol, 1991. **142**(1): p. 23-7.
3. Casey, E., et al., *Genome Sequence of.* Genome Announc, 2017. **5**(23).
4. Chen, B., et al., *Environmental T4-Family Bacteriophages Evolve to Escape Abortive Infection via Multiple Routes in a Bacterial Host Employing "Altruistic Suicide" through Type III Toxin-Antitoxin Systems.* Front Microbiol, 2017. **8**: p. 1006.
5. Denyes, J.M., et al., *The genome and proteome of Serratia bacteriophage η which forms unstable lysogens.* Virol J, 2014. **11**: p. 6.
6. Xing, S., et al., *First complete genome sequence of a virulent bacteriophage infecting the opportunistic pathogen Serratia rubidaea.* Arch Virol, 2017. **162**(7): p. 2021-2028.
7. Jackson, S.A., et al., *Imprecise Spacer Acquisition Generates CRISPR-Cas Immune Diversity through Primed Adaptation.* Cell Host Microbe, 2019. **25**(2): p. 250-260.e4.
8. Matilla, M.A. and G.P. Salmond, *Complete genome sequence of Serratia plymuthica bacteriophage ΦMAM1.* J Virol, 2012. **86**(24): p. 13872-3.
9. Price, L., et al., *Complete Genome Sequence of Serratia marcescens Myophage Moabite.* Microbiol Resour Announc, 2019. **8**(29).
10. Graham, K., et al., *Complete Genome Sequence of Serratia marcescens Phage MTx.* Microbiol Resour Announc, 2019. **8**(25).
11. Campbell, S., et al., *Complete Genome Sequence of.* Microbiol Resour Announc, 2020. **9**(1).
12. Cooper, S., et al., *Complete Genome Sequence of Serratia marcescens Myophage MyoSmar.* Microbiol Resour Announc, 2019. **8**(38).
13. Bockoven, R., et al., *Complete Genome Sequence of Serratia marcescens Podophage Parlo.* Microbiol Resour Announc, 2019. **8**(27).
14. Malone, L.M., et al., *A jumbo phage that forms a nucleus-like structure evades CRISPR-Cas DNA targeting but is vulnerable to type III RNA-based immunity.* Nat Microbiol, 2020. **5**(1): p. 48-55.
15. Melbern, L., et al., *Complete Genome Sequence of Serratia marcescens Podophage Pila.* Microbiol Resour Announc, 2020. **9**(1).
16. Teng, T., et al., *Complete genome sequence analysis of PS2, a novel T4-like bacteriophage that infects Serratia marcescens clinical isolates.* Arch Virol, 2018. **163**(7): p. 1997-2000.
17. Koehler, B.T., et al., *Complete Genome Sequence of Serratia marcescens Siphophage Scapp.* Microbiol Resour Announc, 2019. **8**(19).
18. Williams, E.A., et al., *Complete Genome Sequence of Serratia marcescens Siphophage Serbin.* Microbiol Resour Announc, 2019. **8**(19).
19. Snowden, J., et al., *Complete Genome Sequence of Serratia marcescens Siphophage Slocum.* Microbiol Resour Announc, 2020. **9**(1).
20. Hao, Y., et al., *Complete Genome Sequence of Bacteriophage SM9-3Y Infecting.* Genome Announc, 2018. **6**(1).
21. Recktenwald, J. and H. Schmidt, *The nucleotide sequence of Shiga toxin (Stx) 2e-encoding phage phiP27 is not related to other Stx phage genomes, but the modular genetic structure is conserved.* Infect Immun, 2002. **70**(4): p. 1896-908.
22. Sun, Q., et al., *Isolation and genomic characterization of SfI, a serotype-converting bacteriophage of Shigella flexneri.* BMC Microbiol, 2013. **13**: p. 39.
23. Allison, G.E., et al., *Complete genomic sequence of SfV, a serotype-converting temperate bacteriophage of Shigella flexneri.* J Bacteriol, 2002. **184**(7): p. 1974-87.
24. Mavrodi, D.V., et al., *Mobile genetic elements in the genome of the beneficial rhizobacterium Pseudomonas fluorescens Pf-5.* BMC Microbiol, 2009. **9**: p. 8.
25. Korol, N.A. and F.I. Tovkach, *Identification of the major proteins of the virions of bacteriophage ZF40 Pectobacterium carotovorum.* Mikrobiol Z, 2012. **74**(4): p. 64-70.
26. Paradiso, R., et al., *Complete Genome Sequence of a Myoviridae Bacteriophage Infecting Salmonella enterica Serovar Typhimurium.* Genome Announc, 2016. **4**(5).

27. Mmolawa, P.T., H. Schmieger, and M.W. Heuzenroeder, *Bacteriophage ST64B, a genetic mosaic of genes from diverse sources isolated from Salmonella enterica serovar typhimurium DT 64.* J Bacteriol, 2003. **185**(21): p. 6481-5.
28. George, D.T., et al., *Complete Genome Sequence of SfII, a Serotype-Converting Bacteriophage of the Highly Prevalent Shigella flexneri Serotype 2a.* Genome Announc, 2013. **1**(5).
29. Jakhetia, R., K.A. Talukder, and N.K. Verma, *Isolation, characterization and comparative genomics of bacteriophage SfIV: a novel serotype converting phage from Shigella flexneri.* BMC Genomics, 2013. **14**: p. 677.
30. Juhala, R.J., et al., *Genomic sequences of bacteriophages HK97 and HK022: pervasive genetic mosaicism in the lambdoid bacteriophages.* J Mol Biol, 2000. **299**(1): p. 27-51.
31. Woods, D.E., et al., *Burkholderia thailandensis E125 harbors a temperate bacteriophage specific for Burkholderia mallei.* J Bacteriol, 2002. **184**(14): p. 4003-17.
32. Creuzburg, K., et al., *The Shiga toxin 1-converting bacteriophage BP-4795 encodes an NleA-like type III effector protein.* J Bacteriol, 2005. **187**(24): p. 8494-8.
33. DeShazer, D., *Genomic diversity of Burkholderia pseudomallei clinical isolates: subtractive hybridization reveals a Burkholderia mallei-specific prophage in B. pseudomallei 1026b.* J Bacteriol, 2004. **186**(12): p. 3938-50.
34. Casjens, S.R., et al., *The pKO2 linear plasmid prophage of Klebsiella oxytoca.* J Bacteriol, 2004. **186**(6): p. 1818-32.
35. Zhang, Y., et al., *Lineage and host source are both correlated with levels of Shiga toxin 2 production by Escherichia coli O157:H7 strains.* Appl Environ Microbiol, 2010. **76**(2): p. 474-82.
36. Seed, K.D. and J.J. Dennis, *Isolation and characterization of bacteriophages of the Burkholderia cepacia complex.* FEMS Microbiol Lett, 2005. **251**(2): p. 273-80.
37. Lee, J.H., et al., *Complete genome sequence of Cronobacter sakazakii temperate bacteriophage phiES15.* J Virol, 2012. **86**(14): p. 7713-4.
38. Lee, Y.D. and J.H. Park, *Complete genome of temperate phage ENT39118 from Cronobacter sakazakii.* J Virol, 2012. **86**(9): p. 5400-1.
39. Strauch, E., et al., *Bacteriophage 2851 is a prototype phage for dissemination of the Shiga toxin variant gene 2c in Escherichia coli O157:H7.* Infect Immun, 2008. **76**(12): p. 5466-77.
40. Ogura, Y., et al., *The Shiga toxin 2 production level in enterohemorrhagic Escherichia coli O157:H7 is correlated with the subtypes of toxin-encoding phage.* Sci Rep, 2015. **5**: p. 16663.
41. Villa, L., et al., *Diversity, virulence, and antimicrobial resistance of the KPC-producing.* Microb Genom, 2017. **3**(4): p. e000110.
42. Kyle, J.L., et al., *Escherichia coli serotype O55:H7 diversity supports parallel acquisition of bacteriophage at Shiga toxin phage insertion sites during evolution of the O157:H7 lineage.* J Bacteriol, 2012. **194**(8): p. 1885-96.
43. Moreno Switt, A.I., et al., *Genomic characterization provides new insight into Salmonella phage diversity.* BMC Genomics, 2013. **14**: p. 481.
44. Yang, Z., et al., *Complete genome analysis of bacteriophage AsXd-1, a new member of the genus Hk97virus, family Siphoviridae.* Arch Virol, 2018. **163**(11): p. 3195-3197.
45. Chen, Y., et al., *Isolation and characterization of a novel temperate bacteriophage from gut-associated Escherichia within black soldier fly larvae (Hermetia illucens L. [Diptera: Stratiomyidae]).* Arch Virol, 2019. **164**(9): p. 2277-2284.
46. Bleriot, I., et al., *Genomic analysis of 40 prophages located in the genomes of 16 carbapenemase-producing clinical strains of.* Microb Genom, 2020.
47. Chen, Y., et al., *Specific Integration of Temperate Phage Decreases the Pathogenicity of Host Bacteria.* Front Cell Infect Microbiol, 2020. **10**: p. 14.
48. Zoledowska, S., et al., *High genomic variability in the plant pathogenic bacterium Pectobacterium parmentieri deciphered from de novo assembled complete genomes.* BMC Genomics, 2018. **19**(1): p. 751.
49. Sandner-Miranda, L., et al., *Complete Genome Sequence of Serratia marcescens SmUNAM836, a Nonpigmented Multidrug-Resistant Strain Isolated from a Mexican Patient with Obstructive Pulmonary Disease.* Genome Announc, 2016. **4**(1).
50. Alabed, D., et al., *Draft Genome Sequence of.* Microbiol Resour Announc, 2019. **8**(3).
51. Li, Z., et al., *Complete Genome Sequences of Two Porcine Enterotoxigenic Escherichia coli Strains.* Genome Announc, 2018. **6**(8).

52. Nicholson, W.L., et al., *Complete Genome Sequence of Serratia liquefaciens Strain ATCC 27592.* Genome Announc, 2013. **1**(4).
53. Caneschi, W.L., et al., *Serratia liquefaciens FG3 isolated from a metallophyte plant sheds light on the evolution and mechanisms of adaptive traits in extreme environments.* Sci Rep, 2019. **9**(1): p. 18006.
54. Stein, M., et al., *Complete Genome Sequence of Tetracycline-Resistant Serratia liquefaciens S1, Isolated from Mixed Greens, Obtained Using Illumina MiSeq and Oxford Nanopore MinION Sequencing.* Microbiol Resour Announc, 2020. **9**(19).
55. Sakuraoka, R., T. Suzuki, and T. Morohoshi, *Distribution and Genetic Diversity of Genes Involved in Quorum Sensing and Prodigiosin Biosynthesis in the Complete Genome Sequences of Serratia marcescens.* Genome Biol Evol, 2019. **11**(3): p. 931-936.
56. Weise, T., et al., *VOC emission of various Serratia species and isolates and genome analysis of Serratia plymuthica 4Rx13.* FEMS Microbiol Lett, 2014. **352**(1): p. 45-53.
57. Siwek, W., et al., *Crystal structure and mechanism of action of the N6-methyladenine-dependent type IIM restriction endonuclease R.DpnI.* Nucleic Acids Res, 2012. **40**(15): p. 7563-72.
58. Okwori, A.E., et al., *Pathogenic Yersinia enterocolitica 2/O:9 and Yersinia pseudotuberculosis 1/O:1 strains isolated from human and non-human sources in the Plateau State of Nigeria.* Food Microbiol, 2009. **26**(8): p. 872-5.
59. Roach, D.J., et al., *A Year of Infection in the Intensive Care Unit: Prospective Whole Genome Sequencing of Bacterial Clinical Isolates Reveals Cryptic Transmissions and Novel Microbiota.* PLoS Genet, 2015. **11**(7): p. e1005413.
60. Milne, I., et al., *Tablet--next generation sequence assembly visualization.* Bioinformatics, 2010. **26**(3): p. 401-2.
61. Milne, I., et al., *Tablet: Visualizing Next-Generation Sequence Assemblies and Mappings.* Methods Mol Biol, 2016. **1374**: p. 253-68.
